# Measuring client experiences in long-term care in the Netherlands: a pilot study with the Consumer Quality Index Long-term Care

**DOI:** 10.1186/1472-6963-10-95

**Published:** 2010-04-12

**Authors:** Mattanja Triemstra, Sjenny Winters, Rudolf B Kool, Therese A Wiegers

**Affiliations:** 1 NIVEL, Netherlands Institute for Health Services Research, Otterstraat 118-124, 3513 CR Utrecht, The Netherlands; 2 Prismant, Papendorpseweg 65, 3528 BJ Utrecht, The Netherlands

## Abstract

**Background:**

This study aims to describe the development, testing and optimization of a new standard instrument, the Consumer Quality Index (CQ-index^®^) Long-term Care, for measuring client experiences with long-term care in the Netherlands.

**Methods:**

Three versions of the CQ-index questionnaires and protocols for study sampling and data collection were developed, designed for interviews with residents of nursing or residential care homes and postal surveys among representatives of psychogeriatric residents and homecare clients. From July to November 2006 a pilot study was conducted among 2,697 clients of 68 nursing or residential care homes, 2,164 representatives of clients in 57 psychogeriatric care institutions, and 1,462 clients of 19 homecare organizations. We performed psychometric analyses and descriptive analyses, and evaluated the pilot study.

**Results:**

The pilot study showed the feasibility and usability of the instruments, supported the multidimensionality of the questionnaires and showed first findings on client experiences and possibilities for quality improvement. Nine scales applied to all care settings: shared decision making, attitude and courtesy, information, body care, competence and safety of care, activities, autonomy, mental well-being, and availability of personnel. The pilot resulted in three optimized questionnaires and recommendations for nationwide implementation.

**Conclusions:**

The CQ-index^® ^Long-term Care provides a good basis to investigate the quality of nursing homes, residential care homes and homecare from the clients' perspective. This standardized instrument enables a nationwide comparison of the quality of long-term care for the purpose of transparency and quality assurance.

## Background

The opinions and experiences of consumers in healthcare are generally considered to be relevant indicators of quality of care in addition to indicators used to evaluate the effectiveness, efficiency and safety of care. With a growing demand for patient centered care and for the transparency and accountability of healthcare performance, client surveys are increasingly mandatory for the purpose of public reporting, quality assurance and governance. But the aim, scope, topics and way of questioning of these surveys may vary widely, thus hampering a systematic comparison of healthcare sectors and providers, nationwide benchmarking and monitoring of the quality of care over time.

Therefore, in 2006 the Dutch Ministry of Health, Welfare and Sport mandated the development of a national standard for the measurement and comparison of consumer experiences in healthcare, called the Consumer Quality-index or CQ-index^®^. This standard is based on the American CAHPS^® ^questionnaires [1] and Dutch QUOTE (QUality Of care Through the patient's Eyes) instruments [2-4]. As a registered trademark the CQ-index^® ^is owned by the Dutch Centre for Consumer Experience in Health Care [5] that coordinates the development of CQ-index questionnaires and the conduct of client surveys by certified organizations according to specific guidelines [4].

The new instruments should provide valid, reliable and comparable information about client experiences and their preferences to evaluate the quality of care from the consumers' perspective. Care providers can use this information for quality improvement and for external accountability and public reporting. Results can also be used by: a) consumers to select a health insurer or a care provider; b) client organizations for advocacy services; c) insurers to purchase good care; d) the Health Care Inspectorate and the Dutch Care Authority to supervise and regulate care; e) the Ministry of Health, Welfare and Sport to monitor healthcare.

So far, more than twenty CQ-index^® ^instruments have been developed or are under construction; for health plans [6], for specific sectors or services (primary care, mental healthcare, hospital care and specific surgery) [7-9] and for specific patient groups [10,11].

This article describes the development, testing and optimization of a new sector-specific instrument, the CQ-index^® ^Long-term Care [12]. In the Netherlands, long-term care is generally provided in nursing homes or residential homes (either in somatic or psychogeriatric wards or care units) and in a homecare setting. First findings and experiences with the toolkit (i.e. questionnaires and protocols for sampling and data collection) are presented and evaluated, aimed to assure the usability and feasibility of the instruments for national implementation as standard client surveys in the nursing and care sector. Research questions are:

1) What are the psychometric properties of the draft versions of the questionnaires?

2) What are the experiences with the care provided and what are possibilities for quality improvement?

3) What are the first experiences with the application of the CQ-index and how could the questionnaires and corresponding protocols be optimized?

## Methods

### Development of the CQ-index^® ^Long-term Care

A national Quality Framework Responsible Care for the sector Nursing, Care and Homecare [13] provided a conceptual basis for the CQ-index^® ^Long-term Care (see Additional file 1). This framework represents a nationwide consensus of all parties or stakeholders involved in the sector (i.e. organizations of clients, professionals and care entrepreneurs, the health care inspectorate, care insurers and the ministry of health) on indicators for ten quality domains: Care/life plan, Communication and information, Physical well-being, Safety of care, Domestic and living conditions, Participation and autonomy, Mental well-being, Safety of living environment, Sufficient and competent personnel, and Coherence in care. Each domain includes a set of indicators reflecting the structure, process and outcomes of care [14]. The performance of care providers could be measured either by institutions themselves (i.e. indicators registered at the organizational and client level, for example with the established Resident Assessment Instruments), or by client surveys. For the latter purpose, first a CQ-index had to be developed.

Given the various client populations and domestic settings, three versions of the CQ-index questionnaire and tailored survey methods were designed: a) a questionnaire for face-to-face interviews with residents of somatic wards of nursing or residential homes who were unlikely to fill out lengthy questionnaires because of illness or disability; b) a mail questionnaire for representatives (spouses or family members) of residents of psychogeriatric wards who are unable to participate because of cognitive impairments; and c) a mail questionnaire for clients in homecare who were most likely to be able to complete a self-administered questionnaire.

For each survey setting detailed protocols were developed to ensure standardization of the data collection, i.e. instructions for the selection and sampling of study populations and procedures for conducting the interviews and postal surveys.

To construct draft versions of the questionnaires, relevant questions on the indicators of the national quality framework [13] were selected from existing validated questionnaires on the quality of care and quality of life of residents or homecare clients [15-18] and the CAHPS^® ^Nursing Home Survey [19,20]. Initially, the input from focus groups with clients was used to develop these instruments. Furthermore, results were used from a study on developing quality report cards for long-term care by means of focus groups and concept mapping [21].

Consensus on the selection of items was reached with members of the Steering Committee Responsible Care (i.e. stakeholders, including client representatives). Questions on opinions or satisfaction were reformulated to assess actual experiences, in line with the standard CQ-index format and because experience measures are known to be less subjective and to yield more detailed information for quality improvement [22]. Questions on psychogeriatric care were only formulated for situations that family or other representatives could actually have observed or experienced themselves (i.e. no proxy-ratings). Examples of Experience questions are: "Do caregivers treat you/the client with courtesy and respect?" (Never/Sometimes/Usually/Always) and "Do you have a contact person in the care institution?" (Yes/No). To measure respondents' overall assessment of the healthcare organization and the staff, two global ratings were included. An example of an overall assessment is: "How would you rate the caregivers?" (0 'Worst caregivers possible' - 10 'Best caregivers possible'). Finally, questions on background characteristics of respondents (e.g. age, sex, type and duration of care, self-reported health) were added. This resulted in three draft versions of so called Experience questionnaires. These lists consisted of 83, 76 and 117 questions respectively to measure the experiences of residents, representatives and homecare clients.

For each Experience question a corresponding Importance question was formulated to assess the importance clients attach to different aspects of care (1 'Not important at all' to 4 'Extremely important'). For example: "How important is it for you that caregivers treat you/the client with courtesy and respect?". This resulted in corresponding Importance questionnaires for the three study populations.

### Pilot study

The draft questionnaires and the protocols for sampling and data collection were tested in a pilot study conducted between July and November 2006 in the Netherlands. Four independent research organizations were responsible for data collection. An instruction meeting was organized to ensure consistency of research methods. The registration of consecutive steps of the study sampling together with the experiences of the organizations and their interviewers and the responses of clients enabled an extensive evaluation of the pilot and guided the revision of the instruments. Revisions were made after consultation of the stakeholders.

A total of 144 institutions were recruited through the Dutch organization for care providers (ActiZ) all of which voluntarily participated, yielding 186 locations or wards as units for analysis (Table 1). The participating institutions were randomly divided among the research organizations. Every location or unit was asked to assign a coordinator for the survey and to provide an update client list for the study sampling.

**Table 1:** Number of participating institutions and locations, and sample sizes for each study setting

	Nursing or residential care homes		Homecare
	Somatic care	Psychogeriatric care	
**Participating institutions:**			
Organizations	68	57	19
Locations/wards/units	92	75	19
			
**Sample sizes per location:**			
Experience questionnaire (n)	30	60	200
Importance questionnaire (n)	5	10	20
Total sample required (n)	35	70	220

Exclusion criteria were defined beforehand to increase the homogeneity of study samples within the specific care settings and across units of analyses, and to prevent extra burden of the survey being imposed on the severely ill. A selection was made in cooperation with the nursing staff because they knew about the health and residential status of their clients. Exclusion criteria for the three research settings were:

a) Residential care on somatic wards: residents who were recently admitted (less than one month ago), clients for rehabilitation or reactivation, residents with severe cognitive or psychiatric problems, or residents who were very ill or in a terminal phase.

b) Psychogeriatric residential care: clients with a short stay (less than one month) and residents with a very bad health status or those who received terminal care.

c) Homecare: clients aged under 18 years and those who had received homecare for less than six months.

To enable non-response analyses and to check whether the samples were representative, the total number of clients, the numbers excluded and the reasons for exclusion were registered and client characteristics (gender and age) were gathered for all potential participants.

Because of the length of the questionnaires it was decided to keep the Experience questionnaire and the Importance questionnaire separate and present them to different study samples. The sample sizes (see Table 1) were based on previously applied survey methods in The Netherlands [15-18,23], the CAHPS Nursing Homes field study [20] and expected response rates to the postal surveys (at least 50%). Also practical considerations such as mean number of residents or clients per unit and costs of face-to-face interviews played a role. Relatively large samples of homecare clients were drawn, with equal numbers of clients being selected for domestic care and nursing care, in order to enable the comparison of two types of homecare. Sample sizes for the assessment of importance ratings were much smaller because the variation in answers is known to be small.

Ethical approval of the study was not necessary as research by means of interviews or surveys that are not taxing and or hazardous for patients (i.e. the once-only completion of a questionnaire containing questions that do not constitute a serious encroachment on the person completing it) is not subject to the Dutch Medical Research Involving Human Subjects Act (WMO). Subjects were free to respond to an interview or questionnaire, they were informed about the aim of the survey and they were entitled to stop participating at any time during an interview.

#### Interviews

The research organizations were responsible for training their interviewers, facilitated by an interview protocol on how to prepare, introduce, conduct and finish the interview. For every interview a special form had to be filled out by the interviewer to register the unique codes of the interviewer and the respondent, the number of efforts to make contact, the date of the interview, details about the progress of the interview such as the duration and reasons for breaking it off, comments on difficult questions or problematic answering categories and additional observations.

#### Mail surveys

The mail surveys included two reminders: a thank-you card after one week (in week 2) and a reminder letter with another questionnaire in week 5. A unique identification number enabled the identification of non-responders and non-response analyses. Questionnaires could be sent back to the research organizations in a prepaid envelope. A help-desk was available for phone calls and e-mails about the survey.

### Analyses

First, psychometric analyses were conducted to assess the appropriateness and validity of items and the dimensional structure of the questionnaires. These analyses, also described in the Manual for developing CQ-index instruments [4], included item analyses (percentage of missing responses, skewness, inter-item correlations and importance ratings), explorative factor analyses (Principal Component Analysis with oblimin rotation; Eigenvalue>1; KMO > 0.60 and Bartlett's test of sphericity: p < 0.05) and reliability analyses (Chronbach's alpha for internal consistency of scales). In classical test theory an alpha of 0.7 or higher is recommended for a set of items to be considered a reliable scale [24], but 0.6 is generally accepted as a minimum value in exploratory analyses [25] and we provisionally accepted scales with an alpha between 0.6 and 0.7

Secondly, Experience, Importance and Improvement scores were assessed to get a first impression of clients' experiences and preferences and to determine priorities for quality improvement. Experience scores were calculated for the scales of the Experience questionnaire, with a possible range of 1 (Never/No) to 4 (Always/Yes). Importance ratings were based on the average scores on the Importance questionnaire (1 'Not important' to 4 'Extremely important'). Improvement scores were computed by combining the reported experiences and importance ratings with the formula: proportion negative experiences (Never/Sometimes or No) × Importance score. These improvement scores could vary between 0 and 4, with higher scores indicating a stronger need for quality improvement.

Finally, additional item-analyses were done to optimize the CQ-index^® ^Long-term Care. The aim was to select only relevant, valid and reliable questions. Items candidate for modification or exclusion were selected according to the following criteria:

1) item non-response: >25% answers are missing or item is not applicable (then the number of cases per unit would be too small to compute reliable scores);

2) item skewness: >80% of answers in an extreme 'positive' response category (indicating low variation between cases and settings);

3) item overlap: Pearson correlation between items >0.70 (indicating more than 50% overlap in answering patterns and suggesting that one of these items is redundant);

4) item not fitting in a scale or not attributing to scale reliability: factor loading <0.40 or alpha increases if item is deleted (i.e. item does not contribute to a homogeneous set of items for which a reliable composite score can be computed); and

5) low importance rating for the quality aspect: >25% answered 'not important' (i.e. item does not add much to the content or face validity of the questionnaire).

Items meeting one or more criteria were discussed and modified or deleted after a final discussion with stakeholders (i.e. members of the Steering Committee).

Furthermore, the experiences and comments of respondents and interviewers were used to optimize the order of sections, the wording or clarification of items and the response scales.

## Results

### Survey data

#### Response and client characteristics

Table 2 summarizes the response and client characteristics for each survey setting and each type of questionnaire.

**Table 2:** Response and client characteristics per setting and type of questionnaire

	Somatic care (Residents)		Psychogeriatric care (Representatives)		Homecare (Clients)	
	**Experience**	**Importance**	**Experience**	**Importance**	**Experience**	**Importance**

Number of clients selected (n)	2450	315	2575	233	2599	204
Number of participants (n)	2386	311	2000	164	1363	99
Response (%)	97.4	98.7	77.7	70.4	52.4	48.5
						
Sex: female (%)	73.4	73.6	77.6	74.7	79.3	81.4
						
Age: mean (years)	82.8	82.3	90.2	83.5	76.7	76.9
						
Educational level (%):						
- none or primary education	49.3	54.8	46.5	43.8	33.7	40.2
- secondary or higher education	49.3	44.2	51.0	53.1	63.5	56.5
- other/unknown	1.4	1.0	2.5	3.1	2.8	3.3
						
Duration of stay/care (%):						
- less than one year	26.8	27.2	26.3	33.1	23.5	30.9
- 1 to 5 years	52.9	52.8	60.3	56.5	51.2	33.0
- more than 5 years	20.3	20.0	13.4	10.4	25.3	36.1

#### Interviews with residents (somatic care)

Of the approximately 6.700 residents of the 92 somatic wards, 29% were not eligible to participate in the pilot because of various reasons: cognitive impairments (35% of the excluded residents had severe problems with memory or concentration), too short a stay or rehabilitation (19%), severe illness or terminal care (13%), severe psychiatric problems (12%) or other reasons (21%) such as sensory impairments or other disabilities.

A total of 2,765 residents were selected and invited for an interview and 2,697 (98%) responded positively. Interviewees' were representative of the total eligible population with respect to age and sex (83 years, 74% female).

Eight percent of the interviews stopped prematurely (5% soon after the first questions and 3% halfway), mainly due to cognitive or physical impairments such as memory or concentration problems and fatigue. The mean interview duration was 44 minutes (range 13-100 minutes).

#### Questionnaires for representatives (psychogeriatric care)

The participating psychogeriatric institutions counted on average 60 residents of whom 6% was excluded for various reasons: too short a stay or temporary care (52% of the excluded residents), terminal or palliative care (12%) or other reasons (36%) such as having no relative to fill out the questionnaire.

A total of 2,808 questionnaires were sent to representatives of psychogeriatric clients, and 2,164 responded (77%). The characteristics of the residents to whom the questionnaires referred were fairly similar to the total psychogeriatric population (84 years, 75% female).

#### Questionnaires for homecare clients

The 19 homecare institutions counted on average 1,752 clients and 18% of their clients was excluded, mainly because their care period was too short (96% of the excluded cases had received less than six months homecare, 2% was aged under 18 and 2% was excluded for other reasons such as hospital admission).

Of the 2,803 questionnaires sent 1,613 completed lists returned, and after excluding 151 questionnaires that were not answered by the client (as someone else gave the answers) the response was 52%. Respondents' mean age (77 years) equaled the total client population, but women were overrepresented in the response group (79% versus 70% of all clients).

#### Scales of the questionnaires

Table 3 shows the results of the factor and reliability analyses for the three Experience questionnaires. Explorative factor analyses of the interview questionnaire for residents of somatic wards yielded 18 factors (explaining 58% of the variance), but two factors concerned only single items and some factors showed a similar content (with same items loading on them). Reliability analyses showed that the interview questionnaire comprised seven reliable scales (Cronbach's alpha 0.70-0.83), five scales with a questionable reliability that were provisionally accepted (alpha 0.64-0.69), and three factors that formed no reliable scale (alpha < 0.60). A similar factor structure was found for the questionnaire on psychogeriatric care, and reliability analyses showed 12 consistent scales and one scale that was provisionally accepted (Mental well-being: alpha = 0.60). Finally, the homecare questionnaire comprised 14 reliable scales and also a scale on Mental well-being that was provisionally accepted (alpha = 0.64).

**Table 3:** Scales of the three questionnaires: topics, number of items and reliability (Cronbach's alpha) of the scales

	Somatic care (residents)		Psychogeriatric care (representatives)		Homecare (clients)	
**Scales/themes**	**Items**	**α**	**Items**	**α**	**Items**	**α**

**Care plan and evaluation**	3	0.40*	4	0.44*	5	0.55*
**Shared decision making**	5	0.76	5	0.80	7	0.80
**Communication and information**:						
- Attitude and courtesy of care providers	5	0.83	3	0.75	5	0.78
- Information	7	0.83	6	0.84	6	0.83
- Telephone access/communication	-	-	3	0.70	5	0.83
**Physical well-being:**						
- Body care	4	0.68#	5	0.88	2	0.71
- Meals	5	0.64#	4	0.70	-	-
**Competency and safety of care**:	8	0.82	9	0.89	10	0.86
- Safety of care^§^					5	0.89
- Restraint measures	-	-	2	0.59*	-	-
- Reliability of homecare providers	-	-	-	-	6	0.74
**Living environment**:						
- Comfort	2	0.53*	2	0.53*	-	-
- Atmosphere	4	0.66#	4	0.73	-	-
- Housing and privacy	5	0.69#	4	0.80	-	-
**Participation and autonomy:**						
Activities	5	0.65#	3	0.80	4	0.74
Autonomy	4	0.72	2	0.72	8	0.74
**Mental well-being**	6	0.70	2	0.60#	5	0.64#
**Safety of living environment**	4	0.29*	2	0.54*	8	0.87
**Availability and continuity of care**:						
- Availability of personnel	5	0.74	5	0.86	6	0.72
- Waiting time for homecare	-	-	-	-	3	0.76
- Flexibility of homecare	-	-	-	-	3	0.88

* no reliable scale (α <0,60), thus item scores should be presented separately (no composite scores)

^# ^scales with an alpha between 0.6 and 0.7 were provisionally accepted, but need to be evaluated in future studies

§ extra items on taking care of the clients' health resulted in a separate scale 'Safety of care' for the Homecare questionnaire

The three questionnaires had nine scales in common: shared decision making, attitude and courtesy, information, body care, competence and safety of care, activities, autonomy, mental well-being, and availability of personnel. Six of these scales were sufficiently reliable (alpha 0.72-0.89) and three scales had a lower reliability (0.64-0.69) in at least one setting.

Both the interview questionnaire for residents and the postal questionnaire for representatives contained six items that didn't fit into a scale and that also met at least one of the other criteria for item deletion or adaptation (and three of these items concerned the same quality aspects). The questionnaire for homecare clients counted 11 separate items of which seven were candidate for exclusion or adaptation.

#### Client experiences and opportunities for quality improvement

Homecare clients evaluated their care most positively, with relatively high overall ratings and scale scores (see Table 4). The overall ratings were: 8.36 for the professional caregivers and 8.10 for the institutions. Residential care was evaluated somewhat less positively, with overall ratings between 7.39 (for psychogeriatric care institutions) and 7.97 (for the staff of somatic wards).

**Table 4:** Experiences of residents, representatives and clients: number of valid responses and composite scores* of scales

	Somatic care (residents)		Psychogeriatric care (representatives)		Homecare (clients)	
	**n**	**Mean (s.d.)**	**n**	**Mean (s.d.)**	**n**	**Mean (s.d.)**

Scales (1 'never/no' - 4 'always/yes'):						
**Shared decision making**	2134	2.57 (0.84)	1959	2.74 (0.70)	1267	2.99 (0.68)
**Communication and information**:						
- Attitude and courtesy of care providers	2288	3.38 (0.62)	1930	3.44 (0.55)	1230	3.53 (0.57)
- Information	2108	2.70 (0.63)	1979	3.08 (0.51)	1234	3.12 (0.55)
- Telephone access/communication	-	-	1922	3.34 (0.57)	904	3.33 (0.69)
**Physical well-being:**						
- Body care	1087	3.33 (0.71)	1558	3.03 (0.64)	241	3.40 (0.66)
- Meals	2221	3.43 (0.52)	1643	3.39 (0.52)	-	-
**Competency and safety of care**:	2107	3.35 (0.56)	1781	3.17 (0.51)	1213	3.56 (0.46)
- Safety of care^#^					87	3.48 (0.70)
- Reliability of homecare providers	-	-	-	-	1262	3.72 (0.40)
**Living environment**:						
- Atmosphere	2272	3.23 (0.61)	1912	3.08 (0.56)	-	-
- Housing and privacy	2298	3.59 (0.57)	1955	3.12 (0.86)	-	-
**Participation and autonomy:**						
- Activities	2207	3.25 (0.60)	1690	2.87 (0.71)	932	3.08 (0.75)
- Autonomy	2298	3.44 (0.70)	1087	2.75 (0.95)	1271	3.53 (0.41)
**Mental well-being**	2174	3.11 (0.63)	1285	3.30 (0.61)	1254	3.44 (0.44)
**Safety of living environment**	-^$^	-^$^	-^$^	-^$^	441	3.45 (0.83)
**Availability and continuity of care**:						
- Availability of personnel	2145	2.86 (0.68)	1612	2.93 (0.61)	1130	3.41 (0.55)
- Waiting time for homecare	-	-	-	-	1153	3.45 (0.67)
- Flexibility of homecare	-	-	-	-	537	3.65 (0.86)
						
Overall ratings (0 'worst' - 10 'best'):						
**Care institution**	2255	7.70 (1.32)	1952	7.39 (1.35)	1248	8.10 (1.35)
**Caregivers **(personal care staff)	2229	7.94 (1.24)	1943	7.67 (1.22)	1235	8.36 (1.31)

* mean scores were only calculated if at least one out of every two items of a scale was completed (<50% missings per scale)

# items on Competency and Safety of care form two separate scales care in the Homecare-questionnaire

^$ ^results are not shown because of unreliable scales

Most positive experiences in somatic wards were reported with respect to housing and privacy, autonomy of residents and meals. In psychogeriatric care the attitude and courtesy of caregivers, meals and telephone access and communication scored relatively high. In homecare the reliability, flexibility and competency of the care providers scored best.

Table 5 shows the priorities for quality improvement from the perspective of residents, representatives and clients. While working with a care plan and involving clients turned out to be major targets for quality improvement in somatic wards and homecare, representatives rather emphasized the need to improve the safety of the living environment and the availability of client-centered activities. In residential care, the availability of personnel formed a general concern. In addition, residents highlighted the need for better information, care for their mental well-being and appropriate activities. Relatives also expressed a need to be more involved in decisions about care and restraint measures, as well as more autonomy and better housing circumstances in the psychogeriatric wards. According to the homecare clients, improvements should additionally focus on help to participate in activities, better information, telephone access and communication, and safety of their living environment.

**Table 5:** Priorities for quality improvement, based on quality improvement scores* per theme

Somatic care	Psychogeriatric care	Homecare
1. Care plan and evaluation^# ^(1.53)	1. Safety of living environment^# ^(1.24)	1. Care plan and evaluation^# ^(0.89)
2. Shared decision making (1.36)	2. Activities (1.03)	2. Shared decision making (0.85)
3. Availability of personnel (1.17)	3. Availability of personnel (0.97)	3. Activities (0.67)
4. Information (1.10)	4. Shared decision making (0.95)	4. Telephone access (0.49)
5. Mental well-being (0.76)	5. Autonomy (0.91)	4. Information (0.49)
6. Safety of living environment^# ^(0.71)	6. Housing and privacy (0.72)	5. Safety of living environment (0.48)
7. Activities (0.64)	7. Restraint measures^# ^(0.63)	6. Safety of care (0.47)
8. Comfort^# ^(0.61)	8. Care plan and evaluation^# ^(0.59)	7. Availability of personnel (0.38)
9. Competency and safety of care (0.49)	9. Atmosphere (0.58)	8. Waiting time (0.37)
10. Autonomy (0.48)	10. Information (0.51)	9. Attitude and courtesy (0.32)
11. Attitude and courtesy (0.47)	11. Comfort^# ^(0.47)	10. Flexibility of care (0.30)
12. Meals (0.46)	12. Competency and safety of care (0.44)	11. Autonomy (0.28)
13. Body care (0.43)	13. Mental well-being (0.42)	12. Mental well-being (0.27)
14. Atmosphere (0.42)	14. Telephone access (0.42)	13. Body care (0.24)
15. Housing and privacy (0.35)	15. Meals (0.35)	14. Competency of caregivers (0.22)
	16. Attitude and courtesy (0.32)	15. Reliability of care providers (0.13)
	17. Body care (0.22)	

* Improvement scores (0-4) are reported in brackets. These scores are the product of the proportion respondents (0-1) reporting negative experiences ('never/sometimes' or 'no') and the average importance ratings (1-4) on all items underlying the topic.

# As these themes formed no reliable scales, the reported improvement scores should be interpreted carefully.

### Evaluation of the pilot

#### Sampling

Difficulties encountered in sampling concerned: a) the availability of a digital file with client information in the requested format; b) the risk of bias in the selection of clients; and c) the required sample size if this exceeded the actual number of clients. Thus it was recommended that the study preparations and communication with the participating institutions start early to enable a timely start of the survey, that institutions select their clients in close cooperation with the research organizations, and that more clarity is needed about the sample sizes and the minimal numbers of eligible clients.

#### The interviews

An evaluation of the interviews showed that: a) institutions were hardly prepared for the interviews and often failed to inform their clients, reception and personnel in time; b) there had been insufficient time to train new interviewers; and c) the length of the questionnaire and difficult formulations of items were sometimes problematic. Recommendations were to use a more realistic time schedule, a timely recruitment and training of interviewers, and to use the experiences of interviewers in adapting the questionnaire. Furthermore, a careful selection and training of interviewers and the use of answering cards (showing response options) was recommended to reduce the risk of bias due to interviewer-effects.

#### The mail surveys

Problems and possible reasons for non-response were: a) the length of the questionnaires; b) questionnaires not always tailored to the client's situation; c) the language and wording of questions were not always clear, and d) doubts about the anonymity of the survey. It was recommended to shorten and adapt the questionnaires, and to be open about the privacy protocols used. The problem concerning the 'fit' of the questionnaire was expected because, especially in homecare, the care provided is diverse and often very specific, so that clients do not necessarily recognize all topics of the questionnaire.

#### Revision of the questionnaires

The questionnaires were revised based on the results of the item-analyses and the recommendations of respondents, interviewers and stakeholders. For the Experience questionnaires this resulted in a reduction in the number of questions and the adaptation of many items. The questionnaire for interviews on somatic wards in nursing or residential homes was reduced from 83 to 81 items, and 44 questions were somewhat adapted by rephrasing, adding examples or changing answering categories (e.g. by adding 'I do not know'). The questionnaire for representatives of psychogeriatric clients was reduced from 76 to 72 items and 14 questions were reformulated. The revised homecare questionnaire consisted of 96 instead of 117 questions, including 17 adapted items. For an overview of questions of the revised instruments per quality theme, see Additional file 1: Quality Framework Responsible Care (Appendix 2, part II). The revised instruments and instructions (in Dutch) can be found on http://www.centrumklantervaringzorg.nl/vragenlijsten/verpleging-verzorging-en-thuiszorg.html.

## Discussion

The development of the CQ-index^® ^Long-term Care resulted in three feasible Experience questionnaires with corresponding Importance questionnaires and usable protocols for sampling and data collection in three study populations (i.e. residents of somatic wards, representatives of psychogeriatric clients, and homecare clients). The field test was conducted in 2006 among a total of 6,323 clients (or representatives) of 144 care organizations. The measurement instruments represented the various domains of the quality framework for long-term care in the Netherlands. First measurements gave insight into the quality of care and the opportunities for improvements from the clients' perspective. The pilot study also resulted in recommendations for a nationwide implementation of the instruments for comparative studies among nursing homes, residential homes and homecare institutions.

### Response and general use

In general, clients or their representatives were cooperative and willing to report their experiences. Thereby, it was useful and efficient to have exclusion criteria for the target populations. Particularly in the interview setting the pre-selected sample and the face-to-face situation resulted in a high response rate (98%). The postal surveys among representatives of psychogeriatric clients and among homecare clients resulted in lower response rates (77% and 52% respectively), but also lower percentages of clients were excluded beforehand. For general use, the surveys may well reflect the experiences with long-term care because of the high percentages of eligible cases in each setting (71%, 94% and 82% respectively), the satisfying response rates and the finding that respondents' demographics equaled the populations of interest. Nevertheless, results for the homecare setting must be interpreted carefully while this setting yielded the lowest response rate, with men being underrepresented in the response group, so these results might be biased due to non-response. As a considerable part of non-response in homecare clients might be due to physical impairments, frailty and/or length of the questionnaire, the shortening of the questionnaires might have a positive effect on the future response.

### Comparative and future research

The revised instruments include topics that both stakeholders and clients or their representatives have identified as being important and critical in quality of care. The scales with homogeneous item sets can be used to compute composite scores to compare care providers, and corresponding questions and scales for the different survey settings enable comparisons across the settings. However, to compare the performances of care providers a case mix correction is needed, because client populations may differ on characteristics beyond the control of care providers. Education, age, gender and health status are generally regarded as case mix adjusters [26,27]. In-depth analyses of our pilot data (not presented) showed consistent findings with the literature, with older, lower educated, healthier and male clients reporting on average more positive experiences with care. We also found significant relations with the duration and type of care, and the type of representative or person who assisted in filling out the questionnaires (son/daughter or other relative): with a shorter duration, less intense or complex care, and spouses showing more positive evaluations. However, the results presented were not yet case-mix corrected and further research is needed into case mix correction and differences in the quality of care across providers and settings.

Future research should also focus on changes in performances over time, to evaluate whether feedback reports and transparency leads to quality improvements [6]. Furthermore, if the set of instruments is translated and validated for application in other countries, the surveys can also be used for international comparisons. Other self-report instruments on quality of long-term care [19,28-32] are less comprehensive than the CQ-index^® ^Long-term Care and only few instruments focus primarily on client experiences reports rather than satisfaction or opinion ratings. Nonetheless, as the existing instruments often comprise common domains they could also be synchronized - taking into account local differences in client preferences - in order to enable comparative research between countries.

Finally, as the pilot resulted in recommendations for further standardization of the research method (i.e. sampling and conducting the interviews) and adaptation of the questionnaires, researchers will have to keep on evaluating and optimizing the quality measures and instruments. Apart from studying the psychometric properties with classical test theory, cognitive testing and the use of item response theory (i.e. differential item functioning) would be appropriate to test the validity of items in future research. In addition, external validation testing and more research into interviewer-effects (e.g. inter-rater reliability) are needed.

### Implementation

The Dutch Ministry of Health, Welfare and Sport, the Inspectorate of Health Care and the Dutch organization for care entrepreneurs (ActiZ) have embraced the CQ-index^® ^Long-term Care as the standard instrument for measuring quality from the clients' perspective. The CQ-index has been put in the public domain and implemented nationally as part of the Dutch Health Care Transparency Program [33]. Current legislation requires all health care providers to report certain information about the quality of their services. The long-term care facilities in the Netherlands are now obliged to conduct client surveys with the CQ-index every two years. They have to contract a certified research organization to collect data that will be submitted to a central database for nationwide comparisons, benchmarking and public reporting on internet (http://www.kiesbeter.nl). In 2007 and 2008 another 855 care units and about 35,000 clients were involved (see Additional file 2: public version of the report 'The tone is set' for results of these assessments). Although a boost in quality improvements is expected, research still has to show what organizations actually do with the feedback information. A basis for comparative research and quality improvement has now been provided and systematic evaluations should monitor the implementation and its effects.

## Conclusions

The CQ-index^® ^Long-term Care provides a good basis to investigate quality of nursing homes, residential care and homecare from the clients' perspective. The questionnaires covered all domains of a national quality framework and aspects that are important to clients and stakeholders. At present, the instruments are widely adopted and implemented in two-yearly evaluations of the nursing and care sector in The Netherlands for the purpose of external transparency and internal quality assurance.

## Competing interests

The authors declare that they have no competing interests.

## Authors' contributions

MT and TAW were responsible for the design, supervision and analyses of the study. MT is the principal author of the article. SW was involved in data-collection for the pilot study and produced the first draft of the manuscript, in cooperation with TK. All authors contributed to the revision of the article and approved the final manuscript.

## Pre-publication history

The pre-publication history for this paper can be accessed here:

http://www.biomedcentral.com/1472-6963/10/95/prepub

## Supplementary Material

Additional file 1 **Quality Framework Responsible Care**. Background of the national Quality Framework Responsible Care and Appendix 2 (part II) provides an overview of CQ-index questions per quality theme, for the three survey settings Click here for file

Additional file 2 **Public version of the report 'The tone is set'**. Results of the assessments in 2007 and 2008 are shown in this brochure on the report 'The tone is set: a common language for quality.' Click here for file

## References

[B1] CAHPS: Consumer Assessment of Healthcare Providers and Systems http://www.cahps.ahrq.gov

[B2] Sixma HJ Kerssens JJ Van Campen C Peters L Quality of care from the patients' perspective: from theoretical concept to a new measuring instrument Health Expect 1998 1 2 82 95 10.1046/j.1369-6513.1998.00004.x 11281863 PMC5139902

[B3] Sixma HJ Van Campen C Kerssens JJ Peters L Quality of care from the perspective of elderly people: the QUOTE-elderly instrument Age Ageing 2000 29 2 173 178 10.1093/ageing/29.2.173 10791453

[B4] Sixma HJ Delnoij DMJ (eds) [CQI Manual. An instruction for the development and use of Consumer Quality Index (CQI) questionnaires] [In Dutch] 2007 Utrecht: Centrum Klantervaring Zorg

[B5] Dutch Centre for Consumer Experience in Health Care http://www.centrumklantervaringzorg.nl/centre-for-consumer-experience-in-health-care.html

[B6] Hendriks M Spreeuwenberg P Rademakers J Delnoij DMJ Dutch healthcare reform: did it result in performance improvement of health plans? A comparison of consumer experiences over time BMC Health Serv Res 2009 9 167 10.1186/1472-6963-9-167 19761580 PMC2761896

[B7] Arah OA Ten Asbroek AHA Delnoij DMJ De Koning JS Stam PJA Poll AH Vriens B Schmidt PF Klazinga NS The psychometric properties of the Dutch version of the Hospital-level Consumer Assessment of Health Plans Survey instrument Health Serv Res 2006 41 1 284 301 10.1111/j.1475-6773.2005.00462.x 16430612 PMC1681536

[B8] Stubbe JH Gelsema T Delnoij DMJ The Consumer Quality Index Hip Knee Questionnaire measuring patients' experience with quality of care after a total hip or knee arthroplasty BMC Health Serv Res 2007 7 60 10.1186/1472-6963-7-60 17462084 PMC1876799

[B9] Stubbe JH Brouwer W Delnoij DMJ Patients' experiences with quality of hospital care: the Consumer Quality Index Cataract Questionnaire BMC Ophthalmol 2007 7 14 10.1186/1471-2415-7-14 17877840 PMC2093924

[B10] Damman OC Hendriks M Sixma HJ Towards more patient centred healthcare: A new Consumer Quality Index instrument to assess patients' experiences with breast care Eur J Cancer 2009 45 9 1569 1577 10.1016/j.ejca.2008.12.011 19167212

[B11] Zuidgeest M Sixma H Rademakers J Measuring patients' experiences with rheumatic care: the Consumer Quality Index Rheumatoid Arthritis Rheumatol Int 2009 19370351 10.1007/s00296-009-0926-3

[B12] Wiegers TA Stubbe JH Triemstra AHM [Development of a CQ-index for nursing homes, residential care homes, and homecare; quality of care from the perspective of residents, representatives and clients] [In Dutch] 2007 Utrecht: NIVEL http://www.nivel.nl/pdf/Ontwikkeling-van-een-CQ-Index-voor-verpleeg-en-verzorgingshuizen-en-thuiszorg.pdf

[B13] Steering Committee Responsible Care (ActiZ V&VN LOC, NVVA, Sting, IGZ, VWS, ZN) Quality Framework Responsible Care. The vision documents 'Towards standards for Responsible Care' and 'Standards for Responsible Home Care' made operational via a set of indicators and a control model for long-term and/or complex care 2007 Utrecht http://www.zichtbarezorg.nl/mailings/FILES/htmlcontent/VV&T/Kwaliteitskader%20Verantwoorde%20Zorg%20(english).pdf

[B14] Donabedian A Explorations in quality assessment and monitoring. The definition of quality and approaches to its assessment 1980 Ann Arbor, Michigan: Health Administration Press

[B15] Van Beek APA De Boer ME Van Nispen RMA Wagner C [Responsible care and quality of life among clients of nursing or residential care homes: optimization of a measurement instrument; Report part 2] [In Dutch] 2005 Utrecht: NIVEL

[B16] Poortvliet MC Van Beek APA De Boer ME Gerritsen DL Wagner C [Measuring quality of life in clients of elderly care] [In Dutch] 2006 Utrecht: NIVEL

[B17] Stichting Cliënt & Kwaliteit [Everything okay?] [In Dutch] 2006 Utrecht: SCK

[B18] PwC TNO Management Consultants IWS NIVEL Desan [Branch report Z-org benchmark study homecare 2004] [In Dutch] 2005 Utrecht

[B19] CAHPS Nursing Home Survey - Long-Stay Resident Instrument https://www.cahps.ahrq.gov/content/products/nh/NH_Long-Stay_Instrument.pdf

[B20] Cosenza C Fowler FJ Buchanan JL Cleary PD Ding L Nursing Home CAHPS Field Test Report 2006 Boston: University of Massachusetts, Harvard Medical School

[B21] Groenewoud AS Van Exel NJA Berg M Huijsman R Building Quality Report Cards for Geriatric Care in The Netherlands: Using Concept Mapping to Identify the Appropriate "Building Blocks" From the Consumer's Perspective Gerontologist 2008 48 79 92 18381835 10.1093/geront/48.1.79

[B22] Cleary DC Edgman-Levitan S Health care quality: incorporating consumer perspectives JAMA 1997 278 19 1608 1612 10.1001/jama.278.19.1608 9370508

[B23] Arcares [Benchmark Nursing and Residential care homes 2004/2005. Performances of care providers measured. General report] [In Dutch] 2005 Utrecht

[B24] Nunnally J Bernstein I Psychometric theory 1994 New York: McGraw Hill

[B25] George D Mallery P SPSS for Windows Step by Step: A Simple Guide and Reference. 11.0 Update 2003 4 Boston, MA: Allyn & Bacon

[B26] Hall JA Dornan MC Patient sociodemographic characteristics as predictors of satisfaction with medical care: a meta-analyses Soc Sci Med 1990 30 811 818 10.1016/0277-9536(90)90205-7 2138357

[B27] Hargraves JL Wilson IB Zaslavsky A James C Walker JD Rogers G Cleary PD Adjusting for patient characteristics when analyzing reports from patients about hospital care Med Care 2001 39 6 635 641 10.1097/00005650-200106000-00011 11404646

[B28] Home Health Care CAHPS Survey https://www.homehealthcahps.org

[B29] Chou SC Boldy DP Lee AH Measuring resident satisfaction in residential aged care Gerontologist 2001 41 5 623 631 11574707 10.1093/geront/41.5.623

[B30] Castle N Family satisfaction with nursing facility care Int J Qual Health Care 2004 16 6 483 489 10.1093/intqhc/mzh078 15557358

[B31] Malley J Netten A Jones K Using Survey Data to Measure Changes in the Quality of Home Care Analysis of the Older People's User Experience Survey 2006. Discussion Paper 2417/2 2007 Canterbury: Personal Social Services Research Unit, University of Kent

[B32] Geron SM Smith K Tennstedt S Jette A Chassler D Kasten L The Home Care Satisfaction Measure: A Client-Centered Approach to Assessing the Satisfaction of Frail Older Adults With Home Care Services J Gerontol B Psychol Sci Soc Sci 2000 55 5 S259 270 10985297 10.1093/geronb/55.5.s259

[B33] Zorg Zichtbare Dutch Health Care Inspectorate Framework for quality indicators. A framework for the development and management of quality indicators for the Dutch Health Care Transparancy Programme 2009 Den Haag: Health Care Transparancy Programme Bureau http://www.zichtbarezorg.nl/mailings/FILES/htmlcontent/Programma%20Zichtbare%20Zorg/DEF_Framework%20for%20quality%20indicators_EN.pdf

