# Supplementary material for: Measuring client experiences in long-term care in the Netherlands: a pilot study with the Consumer Quality Index Long-term Care
Source: BMC Health Serv Res. 2010 Apr 12;10:95. doi: 10.1186/1472-6963-10-95 (PMC2907762; doi:10.1186/1472-6963-10-95)
Supplement: Additional file 1 — Quality Framework Responsible Care. Background of the national Quality Framework Responsible Care and Appendix 2 (part II) provides an overview of CQ-index questions per quality theme, for the three survey settings [file 1472-6963-10-95-S1.PDF]

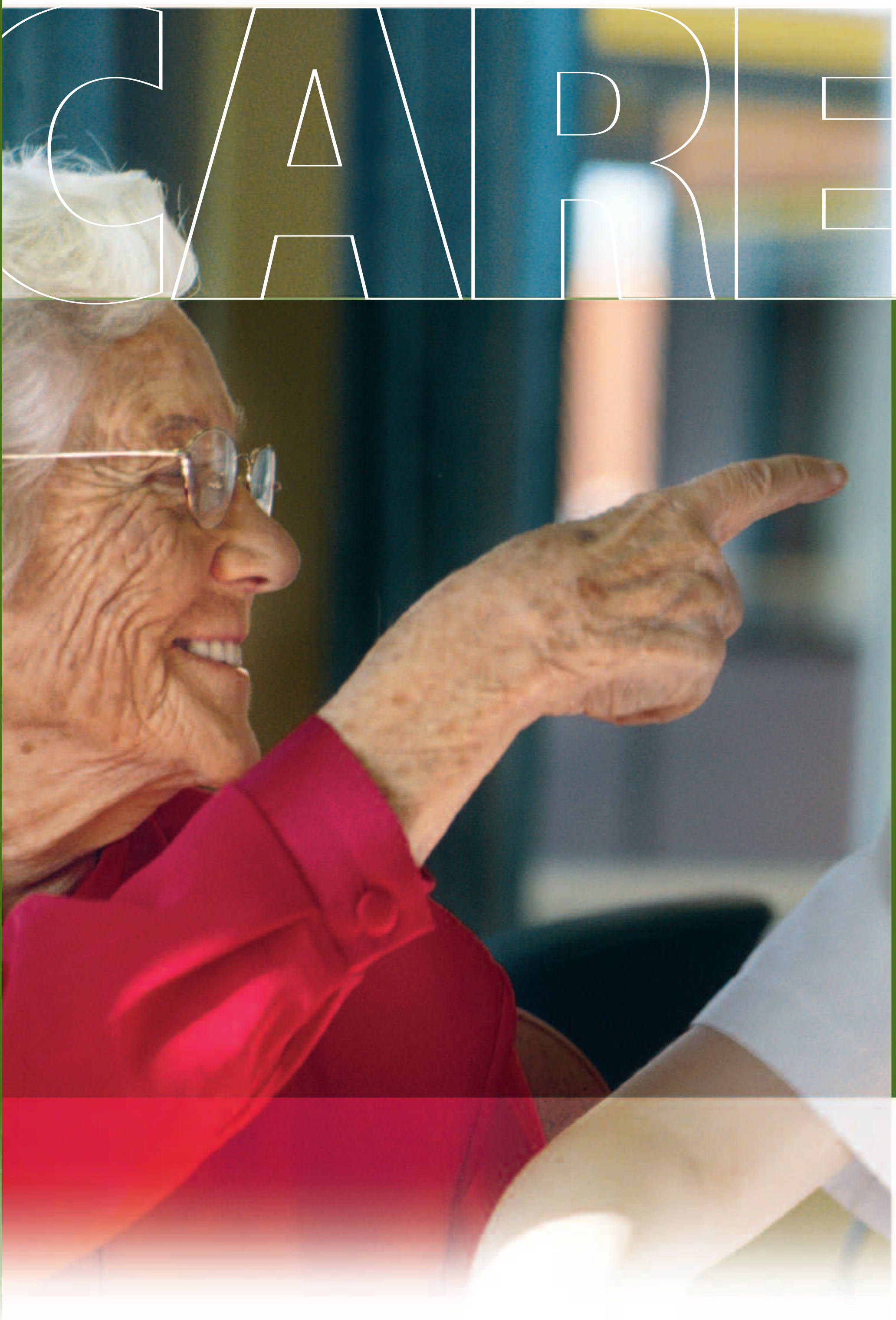

Quality Framework Responsible Care

The vision documents 'Towards standards for Responsible Care' and 'Standards for Responsible Home Care' made operational via a set of indicators and a control model for long-term and/or complex care.

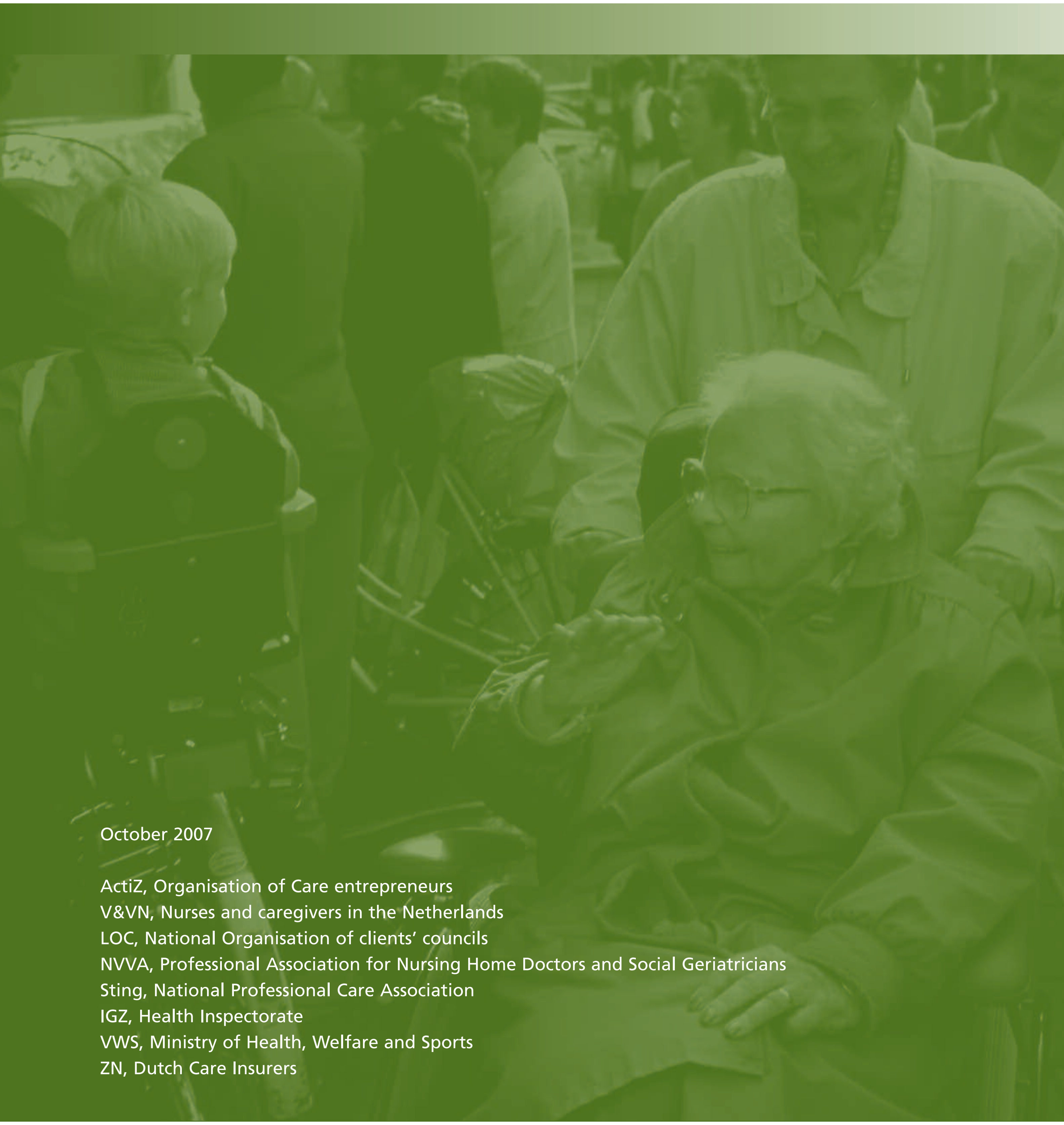

October 2007

ActiZ, Organisation of Care entrepreneurs
V&VN, Nurses and caregivers in the Netherlands
LOC, National Organisation of clients' councils
NVVA, Professional Association for Nursing Home Doctors and Social Geriatricians
Sting, National Professional Care Association
IGZ, Health Inspectorate
VWS, Ministry of Health, Welfare and Sports
ZN, Dutch Care Insurers

Quality Framework Responsible Care

Nursing, Care and Home Care

(Long-term and/or complex care)

The vision documents
'Towards standards for Responsible Care' and
'Standards for Responsible Home Care'
made operational via a set of indicators and
a control model for long-term and/or complex care.

Preface

Below you will find the integrated Quality Framework Responsible Care 2007 for the sector Nursing, Care and Home Care (long-term and/or complex care).
This framework describes exactly all indicators related to care and clients for responsible care as well as a control model which describes how the indicators are used in practice.

The Quality Framework Responsible Care 2007 is based on the Testing Framework Responsible Care. The name has been changed into Quality Framework Responsible Care
to express the fact that we have improved quality here and also to promote a single language within the care sector: also the disabled sector makes use of a Quality Framework. During a pilot project amongst 120 organisations there was a try-out of the Testing Framework 2006. This process has led to the fine-tuning of the indicators. The set of instruments used in order to determine the quality of care is now ready to be implemented; as from May 2007 the Quality Framework Responsible Care has been deployed throughout the sector.

The strength of the Quality Framework is the intensive commitment of all parties involved in the sector. By means of the Quality framework information is collected for various purposes: internal control information for the management, professionals and clients' council, external
accountability information for IGZ and Care Office as well as choice information for consumers.
The single language in the care sector contributes to uniformity and streamlining information and it prevents superfluous doubling of information flows.

The Quality Framework has been established by the Steering Committee Responsible Care which consists of the clients' organization LOC, the professional organizations NVVA, V&VN and Sting, organization of care entrepreneurs ActiZ, IGZ, Dutch Care Insurers and VWS. The Steering Committee is chaired by the Health Inspectorate and is supported by Plexus Medical Group and the Bureau Transparency of Quality in the entire care sector. (ZbTk).

Chair person Steering Committee Responsible Care,

Drs. J.A.H. van Veen
Chief Inspector nursing and chronic care
Health Inspectorate

Contents

**1**	**Introduction**	**5**
	1.1 Standards Responsible Care	5
	1.2 Implementation	5
	1.3 Testing framework 2005	5
	1.4 Pilot Responsible Care	5
	1.5 Quality Framework Responsible Care	6
	1.6 Continuing development	6
**2**	**Starting-points for the set of indicators**	**7**
	2.1 Process or result	7
	2.2 Indicator or standard	7
	2.3 Experiences	8
**3**	**Indicators for Responsible Care**	**9**
	3.1 Themes	9
	3.2 Kinds of indicators	9
	3.3 Background of indicators	10
	3.4 Layered structure	11
	3.5 Measuring	11
**4**	**Control model: working towards quality**	**12**
	4.1 Using information from the Quality Framework	12
	4.2 Quality Framework for internal control and quality policy	12
	4.3 Quality Framework and clients' council	13
	4.4 Quality Framework and layered and phased monitoring	13
	4.5 Quality Framework and care contracting by care agencies	13
	4.6 Coherence between internal control and external monitoring	14
**5**	**Maintenance Quality Framework**	**15**
**6**	**End**	**16**
**References**		**17**
**Appendices**		
	1. 1. Explanation themes Responsible Care VVT	18
	2. 2. Set of indicators for responsible care VVT	21
	I. Explanation set of indicators Responsible care	21
	II. Set of indicators Responsible Care	27
	3. Survey Indicators for Responsible Care VVT	70

1 Introduction

1.1 Standards Responsible care

On 8 June 2005 the organisation of care entrepreneurs ActiZ (previously named Arcares and Z-org), the clients' organisation LOC and the professional organisations AVVV (now V&VN), NVVA and Sting together presented the vision document *'Towards standards for Responsible Care'* (see reference1) to the Parliamentary Under-Secretary.

With this vision document, drawn up in line with the ideas of IGZ, VWS and ZN, a shared picture of responsible care in the intramural nursing-home and residential home care originated. Also for long-term and/or complex care the organisations of care entrepreneurs (Arcares, Z-org, BTN), clients' council (LOC) and professional organisations (AVVV, NVVA, Sting), again in line with the ideas of IGZ, VWS and ZN, have drawn up a vision of responsible care in the document *'Standards for responsible home care'* (see reference 2). Responsible care is good quality care that, at any rate, is given in an effective, efficient, safe and patient-oriented manner and that is attuned to the client's real needs. In the vision document all parties show the willingness to exert themselves in order to realise the goals of the norms for Responsible Care.

1.2 Implementation

In order to help the organisations to provide this 'responsible care', each and every party takes action in connection with their supporters, based on themes shared by all. The execution of these actions will be in line with the programme 'Care for better', which has already started. This programme is executed to the order of VWS by ZonMw (see www.zorgvoorbeter.nl). Organisations that perform well in certain areas are enabled to share their expertise and experience via this programme.

1.3 Testing framework

The professional, care entrepreneurs and clients' organisations mentioned above state in the vision document that the care provided will have to meet field and professional standards and should be guaranteed by an operational quality system. In this way information about quality is sure to be monitored systematically and, if necessary, adapted to structural quality improvement. Furthermore they think it is of utmost importance to check to what extent to care organisations meet the standard for Responsible Care. That is why the focus is on adequate and coherent testing practice that aims at offering ideas for quality improvement. This testing practice was developed in November 2005 for intramural nursing-home and residential home care in the *'Testing Framework for Responsible Care'* (see reference 3) and worked out further for Home Care in the beginning of 2006 (concept not published). The indicators from these testing frameworks made operational what was described as responsible care in the vision documents. It was a deliberate choice to measure results and to name the indicators with a *relative* standard and not to name absolute (minimum) standards (See chapter 2).

1.4 Pilot Responsible Care

On the basis of these testing frameworks a set of instruments has been developed. On the one hand a set of registration questions was developed with the support of Plexus Medical Group that forms the instrument for self-evaluation of care organisations. It is used to measure the so-called *performance indicators concerning content of care*. On the other hand NIVEL has drawn up a set of CQ-index questionnaires to consult clients, with which the so-called *client-bound performance indicators* are formulated.

In a pilot in the summer of 2006 the testing frameworks and the set of instruments going with them were tried out among 120 organisations (see reference 4, 5). It was concluded from the try-out that the instruments generate useful quality information to serve various purposes. The data that is generated fits within the policy context of a receding government authority and itoffers great starting-points for transparency, quality improvement, control and the buying of care to the other field parties. The pilot results, however, led to a fine-tuning of the set of indicators and the registration forms and questionnaires, which has resulted in the current adapted Quality Framework.

1.5 Quality Framework Responsible Care

The Quality Framework Responsible Care contains the standards for Responsible Care in a 'SMART' formulation of a set of performance indicators, adapted on the basis of the pilot experiences. It also contains a so-called control model for the quality framework with a clear division of roles for the parties involved. The accompanying set of instruments (CQ-index VVT questionnaires, Registration forms, Guide lines, Explanation) as well as other information that is important for the measuring of the indicators can be found on or via the website Zorg voor Beter (www.zorgvoorbeter.nl). The set of instruments used to determine the quality of care is now ready for implementation; from May 2007 the' Quality Framework Responsible Care, Nursing Care and Home Care' has been used throughout the care sector.

1.6 Continuing Development

Rather done imperfectly than not done at all

The steering committee Responsible Care starts from a growth model. This means that a technically perfectly validated and 100% reliable measuring instrument cannot be realised immediately. Another point is that there will not be an optimal correction of all relevant factors straight away. Feasibility and efficiency are significant starting-points besides the significance of the indicators. "Rather done imperfectly than not done at all" seems a good summary of this starting-point.

Widening and deepening

A growth model presupposes that the framework can be widened or deepened. In the current quality framework there has so far been no attention for specific categories of clients like rehabilitation clients, clients with non-congenital brain damage or psycho-geriatric clients. Such a focus on target groups (or 'care products') may be desirable in order to make the standards for Responsible Care even more usable for all parties concerned. Clients who only receive domestic support at home and/or Supporting or Activating coaching have not been included in the current quality framework.
In cooperation with, among others, the Association of Dutch Municipalities (VNG) a separate quality framework for domestic care is being developed. Also IGZ, VWS, ActiZ and various organisations of employees and clients are considering the potential development of a quality framework for responsible maternity care.

Evaluation

Finally, the measuring instruments as well as the control model have to be evaluated thoroughly after the first year of implementation to find out in how far they really present a coherent, reliable and valid picture of the quality of care. This, again, may lead to adjustments. The idea is not to make the set larger but to replace, if necessary, certain indicators by others. In the evaluation and the possible adjustments of the CQ-index the Centre Client experience Care (CKZ) plays a decisive role. CKZ controls the brand name CQ-index and, in doing so, sets a number of requirements for the measuring process and the measuring instrument itself.

1 SMART stands for Specific, Measurable, Acceptable, Realistic and Timebound.

2 Starting-points for the set of indicators

The parties involved have used a number of starting-points in the development of the Testing/Quality Framework. You van read up on these in the Testing framework Responsible Care, November 2005 (see reference 3). We shall deal with the most important ones below.

2.1 Process or result

In measuring quality or performance a distinction is made between measuring structure aspects (with the aid of structure indicators), process aspects (with the aid of process indicators) or results (with the aid of result indicators) (see reference 6). An example of a structure aspect is the (infra) structural characteristics of care, like the availability of materials to prevent decubitus. Process aspects in this example would be whether or not to work along a decubitus guide line. An outcome, in this case, would be the number of clients suffering from decubitus ulcers to a certain degree at a certain moment. The starting-point for the quality framework with the standards for Responsible Care is that as much as possible is measured for results. The underlying idea is that how care professionals and organisations provide care should not be reported in detail, but that certain results are realised. Structure or process indicators having a direct relation with the result to be monitored can be applied where there are no result indicators available (or difficult to measure).

2.2 Indicator or standard

In making the standards for Responsible Care operational, indicators have been formulated that cover significant areas of Responsible care. We see the indicator as the 'yard stick' with which we can attain an indication of the quality of care. The indicator does not say what good care is about. To decide what good care is we need to link a standard to the indicator. However, we explicitly propose not to formulate a minimum standard as this has a number of profound disadvantages:

- Minimum standards, in practice, often function as 'maximum standard' (e.g. in discussions on finance), which consequently affects internal control;
- Minimum standards generate futile discussions like "What is a maximally acceptable decubitus percentage?"
- Discussions on minimum standards harm the image of the sector;
- Minimum standards, together with all effects mentioned, are extremely demotivating for professional caregivers and organisations.

That is why the Quality Framework has coupled 'relative standards' to indicating 'best practices'. The scores of care providers (relevantly corrected) are divided in percentiles or quartiles per indicator. This will lead to an average per indicator and a best practice. A target standard can consequently be fixed to this best practice or a best practice from literature. Comparison of every organisational unit with the best practice generates powerful stimuli for improvement. Moreover, working towards maximum quality is good for the image of the sector and does justice to the work of professionals.

2.3 Experiences

The major part of the standards can be tested by asking the clients themselves about their experiences (and not just if they are satisfied). This is first and foremost a fundamental choice: if there is one sector in care where clients' experiences should be the yard stick for determining the quality of care, it is in long-term care. Moreover, literature has shown that asking clients about their experiences delivers a very accurate picture of the quality of care. Finally, this procedure keeps the administrative work load within limits.

3 Indicators for Responsible Care

3.1 Themes

On the basis of both vision documents (see reference 1, 2) one division of themes [2] has been made:

Themes for responsible care			
1.	Care (treatment)/ life plan	6.	Participation
2.	Communication and information	7.	Mental well-being
3.	Physical well-being	8.	Safety living/residence
4.	Safety care content	9.	Sufficient and capable staff
5.	Domestic and living conditions	10.	Coherence in care

Appendix 1 Explanation of themes Responsible Care VVT formulates the content of the themes. There is a short explanation per area. This explanation is not exhaustive but represents the essence of these themes along the lines of the Steering Committee Responsible Care.

Although the choice was made to divide matters in themes, the parties involved would like to emphasise the importance of mutual coherence between the separate themes and indicators. Also the testing of indicators and the public presentation of performance should emphatically be given in coherence. For example an indicator 'falling incidents' cannot be seen apart from an indicator 'measures restricting freedom of movement' .

3.2 Kinds of indicators

Per theme one or a number of indicators have been named. In Appendix 2 *Set of Indicators Responsible care VVT* the indicators are described elaborately. Three kinds of indicators are distinguished:

1. **Client-bound indicators** that are measured by means of a clients' consultation (CQ-index), by an independent agency
2. a. **Indicators measuring the content of care at the level of organisations,** measured by the organisation itself.
   b. **Indicators measuring the content of care at clients' level,** measured by the organisation itself.

2 The specialisations and adjustments that have been made for extramural/home care can be traced back mainly to the levels of indicators and items. In comparison to previous divisions theme 11, Quality Care has disappeared from the Quality Framework because eventually no indicators have been formulated for it.

Theme		Way of measuring		
		Consultation of client	Self-monitoring organisation	
			Organisation level	Client level
		(1)	(2a)	(2b)
1.	Care(treatment)/life plan	x		
2.	Communication and Information	x		
3.	Physical well-being	x		
4.	Safety care content	x	x	x
5.	Domestic and living conditions	x		
6.	Participation and social handiness	x		
7.	Mental well-being	x		x
8.	Safety living/residence	x	x	
9.	Sufficient and competent staff	x	x	
10.	Coherence in care	x		

Table 3.1 Way of measuring indicators per theme

Appendix 3 *Survey Indicators Responsible CareVVT* contains a survey of the indicators in which you can see at a glance what the way of measuring was and its relevance for the various settings. The data collected from the clients' consultations results in 15 to 18 *client-bound indicators* (the number depends on the target group). The data measured by the organisation itself (2a and 2b) together makes up *the indicators for care content.* For nursing and residential care there are 18 and for home care there are 10 indicators.

3.3 Backgrounds of the indicators

The indicators have been carefully drawn up by the Steering Committee Responsible Care and they have been adjusted on the basis of pilot experience.

Client consultation

The CQ-index Nursing Care and Home Care was developed in the period April 2006-April 2007. It consists of three lists of questions concerning long-term and/or complex care: one for the residents of a nursing home or a residential home care institution, one for representatives of psycho-geriatric residents and one for clients living at home. CQ-index is a registered brand, and its owner is the Centre Client Experience Care. This centre requires certain standards from the CQ-index measuring instruments and the measuring process and accredits measuring organisations that use it. Organisations have an accredited measuring institute carry out a measurement using the CQ-index VVT every two years. CQ-index questionnaires, guidelines and information about accreditation can be found on www.centrumklantervaringzorg.nl .

Measuring content of care at the level of the organisation

Various sources have been used to formulate registration questions concerning content of care at a level of organisations. A number of questions from the Inspection form 2003-2004 was copied (Policy measures restricting freedom of movement, Instructions transfer lifts, Competency reserved treatment) but also recent literature was used (Degree of vaccination of staff). The indicators relating to the availability of nursing staff and doctors have been realised on the basis of consensus in the Steering Committee Responsible Care.

Measuring content of care at the level of the client

For the measuring of content of care at the level of clients registration questions have been drawn up for which various sources have been consulted like the National Prevalence Measuring Care Issues, RaiView, CBO guidelines and expertise from improvement routes from Care for Better. In Appendix 1 the sources are given per indicator.

Prevalence

With a great number of indicators the prevalence of a certain care issue is measured, the number of clients which are faced with a certain care issue at one particular moment. It might well be that the incidence figure brings us closer to what we want to know. Incidence means the number of (new) clients with the care issue during a certain period. We have chosen for this procedure because a prevalence measurement involves a relatively more limited administrative work load and a higher level of reliability of the data. In the future we can decide for which kinds of indicators incidence measurement might be better.

Care dependency

On the registration forms a scale has been included to measure care dependency, the Care Dependency Scale (see reference 7). It is of utmost importance to correct a number of indicators for care dependency, so that reliable comparisons are made between organisations. Research is being done to see if the care dependency scoring list, developed for the care dependency packages (ZZPs), can be used for this purpose in future.

3.4 Layered structure

The set of indicators has a layered structure. The first layer consists of the ten themes like Care (treatment) life plan, Communication and information, etc. The second layer is made up of the indicators themselves (e.g. experienced treatment, availability of medical doctor, decubitus). With the client-bound indicators based on client consultation we have a third layer consisting of loose items from the CQ-index questionnaire.

The first two layers will, if it is methodologically justified, come back in the Annual document Social Responsibility and in www.kiesbeter.nl. Information overload should be prevented, but interested parties should be able to gain insight into performance in each of the three layers. In relation to the third layer (the individual CQ-index items) openness is considered undesirable. No conclusions can be drawn on the basis of one isolated CQ-index question and on top of that it creates the risk of damage for individual clients.

3.5 Measuring

The client-bound indicators are mapped through a random test of the client population. Per list of questions (residents in a nursing home or a residential care centre, representatives of psycho-geriatric residents and clients living at home) it is indicated which clients are excluded from participation and how elaborate the random test should be.

The indicators concerning content of care are measured intramurally with all clients within the selection criteria, in home care a random test is carried out. Guidelines for the random test, the way of measuring, the interpretation of the questions etc. have been developed for both client consultation and indicators concerning content of care. We have at our disposal various supporting documents and instruments. Also see appendix 3 Set of indicators and www.zorgvoorbeter.nl.

4 The control model: working towards quality

4.1 Using the information from the quality framework

The quality framework VVT shows to what extent a care organisation is able to realise responsible care in practice. The quality framework serves various purposes; the results in the form of performance indicators are used to achieve for various goals. Measuring only once for different goals leads to a considerable reduction of administrative work loads. Below you will find an outline of the functions of the quality framework VVT and the involvement of certain parties.

4.2 Quality framework for internal control and quality policy

The care organisation is primarily responsible for realising qualitatively responsible care. The quality framework VVT offers important control information to the management and Supervisory Councils. Besides, it is a major source of inspiration to staff within the teams and departments.

Management

The quality framework provides the management with important control information.
This information is based on two sources.

- With the aid of the client-bound performance indicators the management can see which aspects are appreciated by clients and where there is room for improvement.
- With the aid of the performance indicators concerning content of care the organisation gets ideas for an improvement oriented quality policy.

The care organisation renders account publicly about the quality policy followed by taking over the performance indicators in the Annual Document Social Responsible Care. Besides, organisations publish the outcome of the performance indicators on www.kiesbeter.nl for reliable information of the public and to present a profile of the organisation concerning content.

Professionals

The professionals who work at the departments and in teams play a leading role within the quality framework. They are actively involved in the preparation and execution of measurements. It is crucial that the quality framework forms a part of the primary process and that it is not regarded as an extra burden. It constitutes a major part of the regular work and is in line with the professional ambition of employees to deliver good care.
Results from the quality framework offer a basis for further discussion with professionals. Especially there results can be translated directly into concrete improvements and adjustments of programmes and of the way in which tasks are done. Also the Nurses and the Care Advice Councils (VARs) can play an important role here.

Supervisory Council

The quality framework offers Supervisory Councils the opportunity to test if the Board of Directors is ‘in control’ concerning quality and responsible care. On the basis of results from responsible care Supervisory Councils can enter into discussions about the way in which results are translated into quality policy and improvement, the measures that are taken and the extent to which these improvements are actually achieved. The Supervisory Council also monitors whether the clients’ council and the professionals are systematically involved in effecting improvement routes.

4.3 Quality Framework and Clients' Council

The Clients' council plays a major role concerning responsible care. The clients' council represents the residents and clients concerning the quality policy. This role is secured by law. The clients' council has strengthened rights to give advice in relation to the quality policy to be pursued and also the way in which clients are consulted. Also it has been decided that the clients' council will have at their disposal the outcome of the clients' consultation and of the quality tests.

Dialogue important

The clients' council receives the integral reporting about the clients' consultation, as well as the outcome of the self-evaluation carried out by the institution. The Board of the care organisation always seeks advice from the clients' council concerning their insights and preferences in relation to necessary improvements in quality.
The dialogue between the management and the clients' council is an important key-stone of the quality framework responsible care. The clients' council, if any, is the party that indicates, on behalf of the clients, in what areas there should be improvement of quality.

Support clients' council

A great part of the clients' councils is a member of the LOC and has therefore the possibility to be supported by a LOC-advisor. If this is the case, the advisor will be briefed beforehand by the management about the plan to carry out a clients' consultation and gives the management the disposal of the outcome of the consultation and the self-monitoring. In this way the advisor is well able to support the clients' council in the dialogue with the management about the outcome of the quality framework.

4.4 Quality framework and the layered and phased supervision

IGZ is responsible for the monitoring of quality care. To this end IGZ uses the information that is given by organisations on the basis of the quality framework Responsible Care. IGZ uses the results from the quality framework to form its judgement on safe, effective and qualitatively responsible care. In the procedure of supervision Layered and phased Supervision (GGT) take a central place. This encompasses the following:

- For stage 1: (determining the risk of irresponsible care) all indicators, based on measurements and client consultation, as has been decided in the quality framework, are taken into account.
- Annually, a percentage is determined where an inspection visit in stage 2 of GGT will take place. This is not just about the high-risk organisations but also about a selection from the middle group and the best practice organisations. If necessary IGZ may decide after the inspection visit to carry out strict control or to proceed to further enforcement (stage 3).
- Finally, IGZ carries out supplementary theme-based research. These themes are determined annually.

4.5 Quality Framework and care contracting by care agencies

To care agencies quality is one of the key-stones in their contracting policy. And quality can most clearly be seen in the performance indicators from the quality framework.
In contracting the care agencies require the organisation to have a working quality monitoring system, as has been laid down in the law pertaining to quality. This quality system is characterised by the following aspects:

- The ten content themes from Responsible Care are central;
- By means of monitoring the indicators concerning content of care, there is an annual, adequately executed, self-evaluation of these themes;

- Every two years there is a clients' experience survey, carried out by means of the nationally accepted tool, the CQ-index;
- The outcomes of both form the basis of an internal improvement cycle, in which priorities are determined in close dialogue with the clients; council.

For 2008 the care bureaus have indicated that they would value an external audit (which would lead to a certificate) of the working quality system, also because an external audit contributes to a thorough self-evaluation and reliable information. Moreover, an external audit creates alertness towards the translation of the outcome of performance indicators into improvement of working processes. Care agencies are of the opinion that any quality system should seamlessly take over the nationally determined set of indicators. On the basis of progressive insight it will be decided in 2009, when the results of performance indicators for all institutions are known, what the external audit should encompass.

4.6 Coherence between internal control and external supervision

It has been indicated above in which way and from what responsibilities the various parties make use of the same quality framework. It is crucial for those parties to enforce one another and that all interventions contribute to quality improvement.
The Steering Committee has defined the following starting-points for the control model:

- The management discusses the outcome with the clients' council and professionals and draws up agreements about improvement routes and targets. Also it is indicated in what way lessons can be learned from the best practices and break-through projects of the programme Care for Better.
- Targets and actions for improvement are presented to IGZ, if they ask for it. IGZ will judge them within the framework of its supervisory tasks. Its focus will be safety and health risks.
- IGZ carries out inspection visits, especially where risks are involved and monitors factual improvements. IGZ reports are public and can be read on www.kiesbeter.nl .
- The care organisation reports on the outcome and improvement actions in the Annual Document Responsible Care.
- The care bureau weighs the outcome of the quality framework in its contracting policy. The bureau makes use of available quality information and planned routes of improvement from the annual document care, because the quality realised may eventually affect agreements about care production (volume and/or price).

5 Maintenance Quality Framework

The indicators for Responsible Care and the instruments to measure it have been adapted on the basis of the pilot and they have been decided upon for the measuring period April 2007 - April 2008 by the Steering Committee Responsible Care. The instruments need to be maintained and, if necessary, improved, adjusted or fine-tuned. Continuous adjustment to new insights from science, developments in the field, changing needs and perceptions of clients are central to a quality framework as it has been formulated here.

For the maintenance of measuring instruments 'togetherness' is the starting-point. This means that the parties involved will eventually decide on these instruments together. The underlying idea here is that the standards for Responsible Care are a collective responsibility. To do justice to this coherence responsibilities have been divided as follows:

- Proposals for further development and improvement of the indicators are formulated by a Project group in which all relevant parties are represented;
- Determination of the proposals is done by the Steering Committee;
- The professionals and organisations lead the further development and improvement of the indicators concerning care content; all other parties look on and deliver input;
- In the further development and improvement of the client-bound indicators (CQ-index) the clients' organisation leads; all other parties look on and deliver input;
- Determination of the renewed instruments is always a collective responsibility and is done within the Steering Committee;
- IGZ has a special position in all this. Public availability of performance data per organisational unit (ideally at target group level) is a conditio sine qua non for the proper functioning of the new care system. IGZ has the task to a) see to it that this information is in actual fact available and b) to monitor the delivered quality performance by caregivers. Because of this function IGZ has been positioned as producer. It will guard the collectiveness of the development route and sees to it that the testing framework is realised and developed further. On the basis of its legal authorities IGZ can always points of interest or indicators to the Quality Framework. This will, however, always be in line with professional standards and existing literature. IGZ can also independently request additional information from providers, if the Quality Framework is not up to standard (assessed from the point of view of the double task of IGZ as mentioned above).

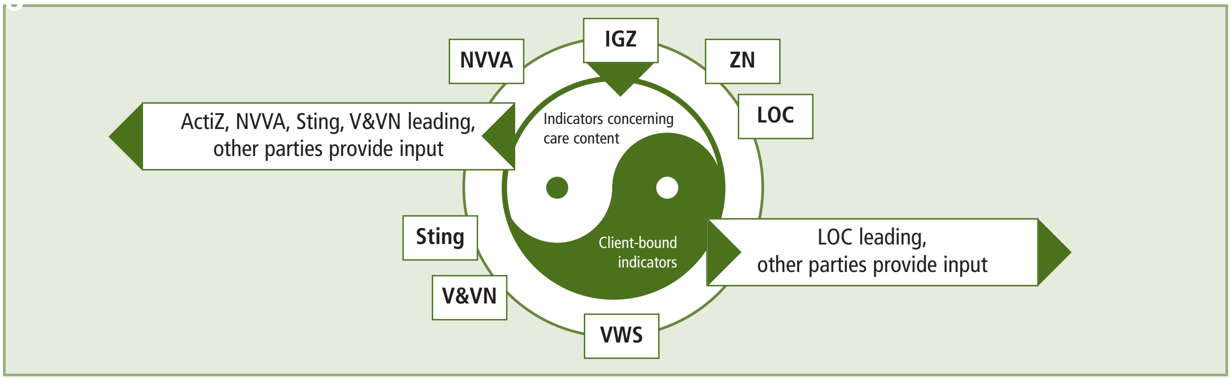

Figuur 5.1 Division of roles for the maintenance and improvement of the indicators concerning care content and client-bound indicators and the instruments belonging to it.

End

From May 2007 the Quality Framework Responsible Care has been implemented at all institutions for nursing, care and home care. This means that they carry out their own yearly registration of the content-related indicators and that they have an external party carry out a clients' consultation with the aid of the CQ-index. In the annual document Socially Responsible Care for 2007 all care institutions from the sector V & VT show themselves to be transparent for the first time as far as the performance indicators described in this document are concerned.
It has become apparent (from the pilot in 120 care organisations) that the Quality Framework constitutes a considerable broadening of the possibilities to control quality. For the institutions themselves (including the Supervisory Councils) and the professionals employed there, the Quality Framework provides a lot of information on what goes well and where quality improvement is necessary. Clients' councils can give excellent advice on the basis of the available information about quality improvements they deem important. Clients receive comparable information in order to make a choice themselves for a good provision of care.
IGZ and insurers gain insight into the actually delivered performance by care organisations and so they can stimulate, reward and monitor quality at a much more differentiated level than before. The balance between quality improvement and external responsibility has appeared to be crucial in this.
Implementation of Responsible Care is a process which is monitored with great care. In 2009, after the implementation in all care organisations, en extensive evaluation will be carried out. This may lead to further adjustment of the set of indicators. New indicators can replace old ones. Besides, additional indicators will be developed for specific target groups.

This means that the standards for responsible care and the quality framework responsible care will remain living 'concepts' , in tune with developments with in the care sector and focussed on the information needs of the management and the Supervisory Councils of care organisations, clients' councils, staff, IGZ, and care insurers. All this has one objective: an on-going process of improvement of quality of care!

Referenties

(1) Arcares, AVVV, LOC, NVVA, Sting, in coordination with IGZ, VWS and ZN (2005). *Towards standards for responsible care; a development model for nursing homes and care institutions drawn up by clients' organisations, providers, professional groups.* Utrecht: Steering Committee Responsible Care.

(2) Arcares, Z-org, BTN, AVVV, LOC, NVVA, Sting, IGZ, VWS and ZN (2006). *Standards for Responsible Home Care; a model for long-term and/or complex home care and for long-term and/or complex extramural care from nursing homes and care institutions, drawn up by Arcares, Z-org, BTN, AVVV, LOC, NVVA and Sting and in coordination with IGZ, VWS and ZN.* Utrecht: Steering Committee Responsible care.

(3) Arcares, AVVV, LOC,NVVVA, Sting, Z-org, IGZ, VWS, ZN (2005). *Testing framework for Responsible Care; Operationalisation of the vision document 'Towards standards for responsible care' in a set of indicators and a control model for the V&V sector.* Utrecht: Steering Committee Responsible Care.

(4) Wiegers, T.A., Stubbe, J.H. and A.H.M. Triemstra (2007). *Development of a CQ-index for nursing homes and care institutions and home care; Quality of care according to the residents, representatives and clients.* Utrecht: NIVEL

(5) Plexus Medical Care (2007). *Evaluation standards for responsible care; Evaluation of the pilot Standards for responsible care in nursing, care and home care (01/01/2006-30/11/2006).* Amsterdam: Plexus Medical Group

(6) Donabedian, A. (1980) *Explorations in quality assessment and monitoring (vol. 1).The definition of quality and approaches to its assessment.* Ann Arbor, Michigan: Health Administration Press, p. 79

(7) Dijkstra, A., Buist, G.A.H., Dassen, T.W.N. and W.J.A. van den Heuvel (1999). Care Dependency Scale (CDS); a guideline. Groningen: Noordelijk Centre of health care issues Rijksuniversiteit Groningen.

Appendix 1: Explanation themes Responsible Care VVT

The Quality Framework Responsible Care VVT has been divided in ten themes. This appendix indicates the content of the themes. Per theme you will find a short explanation below. It is by no means exhaustive but it states the essence of the themes in line with the ideas of the Steering Committee Responsible Care. A more extensive explanation can be found in the vision documents 'Towards standards for Responsible care' and 'Responsible Home Care'.

1 Care (treatment) / life plan

In essence

- every client has an indication decision, a care contract and a care(treatment)/life plan;
- clients are involved in drawing up, evaluation and adjustment of the care(treatment)/life plan;
- the four domains of responsible care are to be found in the care(treatment)/life plan;
- the care(treatment)/life plan gives a picture of care demand, care need and indication;
- rights and duties of clients and care organisation, support of clients in drawing up the care (treatment)/life plan and contracts with third parties (like the general practitioner) have been put down in writing;
- the care contract is in line with the indication.

2 Communication and information

In essence

- communication is open and the care organisations puts efforts into clients' wishes to improve matters;
- clients receive clear and understandable information about rights, the care(treatment)/life plan and the care contract, clients payments, complaint procedures and the clients' councils;
- clients have a regular contact person at the care organisation;
- the way clients are treated is in order;
- representatives and clients living at home can be reached easily by phone.

3 Physical well-being

In essence

- the care organisation provides adequate support with, among other things, washing/showering, dental care, nail care, going to the toilet, use of incontinence products;
- clients are satisfied about the moment care is given and the way in which it is administered;
- meals are tasty in nursing and care centres; there is sufficient staff to support at meal times and the ambiance is cosy.

4 Care-related safety

In essence

- the care organisation provides safe care;
- Issues that are dealt with are: decubitus, unintentional weight loss, fall incidents, incontinence, Foley Balloon catheter and the experienced professionalism/safety of caregivers;
- Besides, specific points of attention in nursing homes and care institutions are: fixation, problematic behaviour, vaccination, medicine-related incidents, under nourishment and psychofarmaca
- In home care organisation there is specific attention for the policy of restriction of the freedom of movement.

5 Domestic and living conditions

In essence

- the care organisation respects the clients' privacy and their own living atmosphere;
- living-space is pleasant in nursing homes and care institutions, the cleaning of the apartments is up to standard and clients have the possibility to furnish their apartments with their own belongings;
- the ambiance of the building is a pleasant one and there are opportunities for mutual contact among residents.

6 Participation and social handiness

In essence

- clients have sufficient opportunities to participate in various activities and to have social contacts;
- there is sufficient help for residents to go somewhere;
- a sufficient number of quality activities are organised for the residents;
- there is attention for support in doing odd jobs (like filling in forms) and the finding of activities;
- the care organisation promotes independence and autonomy as in subjects like determining for oneself how one arranges activities during the day and deciding when one actually wants care to be given.

7 Mental well-being

In essence

- caregivers pay attention to client support in their personal development, choices in life and their sense of purpose;
- there is sufficient attention for clients suffering from loneliness or depression;
- there is sufficient mental care for residents of nursing homes or care institutions;
- changes in the mental well-being of residents are spotted in the home situation and passed on.

8 Safety residence/living-space

In essence

- safety of the residence and living-space is up to standard, like supervision for psycho-geriatric residents and the question if residence know what to do in case of emergencies;
- clients feel safe;
- caregivers spot unsafe situations with clients living at home and consequently they react adequately;
- caregivers are reliable and the handle personal property with care;
- caregivers have been instructed sufficiently how to work with equipment (like transfer lifts)

9 Sufficient and capable staff

In essence

- there is enough staff available;
- enough time is spent on the clients;
- caregivers are capable professionals and reserved treatment will only be administered by qualified staff;
- attention is given to the number of caregivers in the home situation and how replacement of staff is dealt with;
- with function treatment there is a nurse present and a doctor available.

10 Coherence in care

In essence

- coordination takes place with other (care) organisations and caregivers.

This area is still under development. The objective is to arrive at good agreements between the care organisation and other care organisations/caregivers so that the client is not bothered by compartmentalisation within care and closely related sectors.

Appendix 2: Set of Indicators for Responsible Care VVT

Contents

I. Explanation of Set of Indicators for Responsible Care 21

1. A bird's eye view 21

2. Organisational units 22

3. Measuring and registering
   3.1 Client-bound indicators 22
   3.2 Indicators concerning content 24

4 Technical data base 26

5 Publication of performances 26

6. Support 26

II. Set of Indicators for Responsible Care 27

I. Explanation of Set of Indicators for Responsible care

1. A bird's eye view

The set of Indicators belonging to the Quality Framework Responsible Care consists of two parts: the client-bound indicators which a care organisation (an organisational unit) has measured via a clients' consultation and the indicators concerning content of care, measured by the organisation itself, partly at the level of clients and partly at the level of the organisation.

The clients' consultation has to be carried out once every two years by an accredited measuring agency. To this end the CQ-index VVT questionnaires and guidelines have been developed. The indicators concerning content of care are measured every year with established registration questions. The data concerning content of care can be registered in various ways: with the ActiZ registration tool VZ, the national prevalence measurement of care-related issues (LPZ), RaiView, or a registration system or ECD of the organisation itself.

Via the measuring agencies and registration systems the data is combined nationally in a technical data base. Here the data for relevant client characteristics (like care dependency), is corrected and transferred into comparable performance scores. These are fed back to the organisational unit in the form of a report which aims at internal improvement. Moreover, the performance data is fed back in such a way that it can be presented in a user-friendly manner for accountability in the Annual Document Social Responsibility, and as consumer information to www.kiesbeter.nl.

2. Organisational Units

Within the framework of the Quality Framework Responsible Care every organisational unit[1] (OE) has got to give a clear picture of the indicators for Responsible Care. The term and definition 'Organisational Unit', as used by IGZ for the Annual Document Social Responsibility has been chosen. In Intramural care it is often a location and in home care it is mostly a region. IGZ determines the organisational unit. IGZ monitors a list of organisational unit codes (OE codes). This is accessible on www.zorgvoorbeter.nl. Both the clients' consultation and the care

3. Measuring and registering

The quality Framework Responsible Care has ten themes:

1.	Care (treatment)/ life plan	6.	Participation
2.	Communication and information	7.	Mental well-being
3.	Physical well-being	8.	Safety living/residence
4.	Safety care content	9.	Sufficient and competent staff
5.	Domestic and living conditions	10.	Coherence in care

Per theme one or a number of indicators have been mentioned. We distinguish between different kinds of indicators:

1. **Client-bound indicators** that are measured by an independent agency by means of a clients' consultation (CQ-index);
2. **Indicators concerning care content at the level of the client,** measured by the organisation itself (4.1, 4.2a, 4.2b, 4.3, 4.4, 4.5, 4.6a, 4.7a, 4.7b, 4.8, 4.9, 4.10, 7.2)
3. **Indicators concerning care content at the level of the organisation,** measured by the organisation itself (4.6b, 4.11, 8.3, 9.2, 9.3, and 9.4)

The indicators are elaborately described in this appendix. It also contains a survey of the indicators, where you can quickly find the way of measuring and the relevance for the various settings per indicator. The data collected through the clients' consultation (1) result in 15 to 18 *client-bound indicators.* This number depends on the target group involved. The data that is measured by the organisation itself (2a and 2b) together form the *care content indicators.* For Nursing and Care there are 18 (5+13) indicators and for Home Care there are 10 (3+7) indicators.

3.1 Client-bound Indicators

Centre Client Experience Care

The Centre Client Experience Care (CKZ) was founded on 6 December 2006. This organisation sees to it that experiences of consumers related to health care are registered systematically over the next few years and are measured reliably and published.
To this end we have a national standard of measuring, the CQ-index. On its basis an elaborate family of lists of questions is developed. Measurements with the CQ-index provide input for the website

[1] 'An organisational unit is an organisational coherent group that forms part of a holder (care provider) who is legally responsible for giving care. This can be a nursing home, a care institution or a work area. An organisational unit can provide care at more than one location. A location is an establishment as part of the organisational unit. The organisational unit is (financially) responsible for care administered at that particular location.' (IGZ form Layered and Phased Supervision, 2005)

www.kiesbeter.nl, which is a site that helps consumers in making a well-considered choice from different care providers.
For the sector VVT a CQ-index has been developed and CKZ supervises its use as well as the future development of those questionnaires. To meet the standards for Responsible Care every organisational unit should have an accredited measuring agency carry out a clients' consultation every two years with the CQ-index VVT. This agency works in line with the guidelines determined by CKZ. You can find them on www.centrumklantervaringzorg.nl

Target groups

The CQ-index Nursing, Care and Home Care consists of three questionnaires with specific target groups:

1) *CQ-Index Experiences with care in nursing homes or care institutions; a questionnaire for interviews with residents;* this list is meant for clients who stay at a nursing home or care institution for one month and with whom an interview can be carried out. The list is not meant for reactivation or rehabilitation clients.
2) *CQ-index Experiences with care in a nursing home or care institution; questionnaire for representatives of residents;* this list aims at representatives of psycho-geriatric clients who receive care for more than one month. The list is not meant for short-stay clients, respite care and the like.
3) *CQ-index Experiences with home care; questionnaire for clients living at home from home care organisations or care organisations;* this list aims at clients of 18 years old or older who live at home, who receive personal care and/or nursing at home (possibly living next to a care centre) for a period of a minimum of six months.
   This list is not meant for clients who receive domestic care only and/or Supportive or Activating Coaching.
   The CQ-index questionnaires can be downloaded from www.centrumklantervaringzorg.nl.

The moment at which measuring takes place

The clients' consultation is assigned by the care organisations and is carried out at a moment to be decided on by themselves but at least every two years. It is recommended to carry out the measuring of care content and the clients' consultation in the same period. This prevents empty fields on the quality card on www.kiesbeter.nl and gives better opportunities to correlate the measurements because they were carried out in the same period.

Investment

The organisational unit has an independent measuring agency carry out a measurement with the CQ-index VVT. Costs are at the expense of the Organisational Unit.
To have the measurements carried out properly the organisational unit is expected to appoint a contact in the organisation for gearing to one another (not coordinating) the planning and period of research, the information for residents and staff (info meeting, brochures/letters), delivering digital lists of residents for random checks and the approach of residents or their representatives.

3.2 Indicators concerning content of care

Registration forms

On www.zorgvoorbeter.nl registration forms for Nursing home & Care institution care (V&V) and for Home Care (ZT) can be downloaded. There are separate registration forms for V&V and ZT, and for both V&V and ZT there is a registration form at the level of the organisation and one at the level of clients. The registration forms at the level of clients also encompass the necessary factors that are essential for corrections, so that performances can be compared.

Target groups

The group of clients with which measurements are carried out is different for Nursing Home Care & Care Institution Care (V&V) and Home Care.

- *V&V*
  Measuring of indicators concerning content of care at client level is carried out with all clients for V&V, excluding:
  - clients who receive care shorter for fewer than 30 days;
  - clients who receive rehabilitation care, only day activities, respite care and the like;
  - specific groups of clients mentioned by the indicator;

  vclients who do not want to be examined (see ‘refusing to be examined)

- *ZT*
  Measuring of indicators concerning content of care at the level of clients is carried out by ZT through a random choice from the client population, excluding:
  - clients who receive care for fewer than 30 days;
  - clients under 18;
  - clients who only make use of domestic care and/or Supporting or Activating coaching;

  Subsequently in Home Care the following groups are not measured:
  - specific groups of clients mentioned in the indicators;
  - clients who do not want to be examined (see ‘refusing to be examined’)

  For the random check we have a specific key. See table 1 fore an indication of the size of the random check among various groups of clients. At www.zorgvoorbeter.nl you can find a simple tool which will enable you to calculate the size of the random check exactly.

Number of clients	<50	100	200	300	500	750	1000
Size Random Check	alle	83	141	184	244	291	322

Tabel 1 Indication of size of random check of the indicators concerning content of care at the level of clients in Home Care.

Refusing to be examined

With indicators concerning content of care at the level of clients for which examination is necessary (like for example decubitus, problem behaviour, depression), there is the possibility that clients do not want to be examined. This can be due to two situations:

- in the care contract or care(treatment)/life plan) it has been decided in agreement with the client not to systematically examine the client for the benefit of the Quality Framework;
- the client cannot (for example because of illness) or does not want to be examined on the day or in the week of measuring.

The above implies that the procedure of the indicators for Responsible Care (especially the periodical measurements of these indicators) is discussed with every client who receives care from the organisational unit and that it is put down in writing in the care contract or in the care (treatment)/life plan whether or not the client agrees with a periodical examination for the indicators involved for the benefit of the Quality Framework. Should a client renounce periodical screening for a certain aspect of the Quality Framework, then this will be put down in writing with motivation. Agreements about this should be evaluated on a regular basis.
For certain indicators at the level of clients, like fall incidents (4.3), medicine incidents (4.4), psychofarmaca (4.5), degree of vaccination of clients (4.6a), Foley Balloon catheter 4.8), fixation (4.10) this agreement does not apply.

The moment of measuring

The registration questions are filled in during the measuring periods fixed by the Steering Committee. Information about the measuring periods is to be found at www.zorgvoorbeter.nl.

Registration systems

LPZ and Rai-View

Organisations participating in the module Standards for Responsible Care (3N) of the National Prevalence Measurement Care Problems (LPZ), or organisations working with Rai-View can, via that route, measure the indicators concerning content of care (both the indicators at the level of organisation and of clients). Organisations, however, should be aware of the definition of 'organisational unit' (see section 1), so that the data is collected at the correct level of organisation.

Organisations' own registration / ECD

In the long run a more continuous registration of indicators concerning content of care is desired. The data collected provides a lot of information to the professionals and managers involved about care intensity of clients as well as the quality of care delivered. If this is done on a regular basis, it will provide steering information. This is exactly what an instrument like Rai-View does. Also an Electronic Clients' Dossier (ECD) can be set up in such a way that certain aspects are measured as an integral part of the daily working process. This is the situation that we eventually would like to reach. Organisations that can already collect indicators concerning content of care through an ECD or via their own registration system are welcome to link their system to Responsible Care.
For these organisations and their ICT suppliers what requirements the supply of data should meet in order to be accepted in the national data base.

Registration tool VZ

Organisations that cannot yet collect their indicators concerning content of care for Responsible Care from their current care registrations can make use of the free ActiZ Registration tool Responsible Care. This is an instrument with which organisations can register their indicators concerning content of care at the level of clients and at the level of organisations in a period of measuring and deliver the information to Responsible Care.
This tool is meant for an annual measurement and should not be used for continual registration. This registration tool can be used the following three years and by then organisations are expected to have developed their own ECDs or other, more continuous registration systems which are fully integrated in the day-to-day working processes and registrations.

Investment

From the pilot Responsible Care ii has become clear that collecting data at the level of clients takes between 10 to 15 minutes. In order to have measurements taken in a proper manner there should be a coordinator in the care organisation who sees to the coordination of, among other matters, the instruction of caregivers, the planning of measurements at the level of clients, the collection of data at the level of organisations, the handing out of forms, the feeding of data, et cetera.

4. Technical data base

The data from the indicators concerning content of care and the client-bound indicators are collected in two separate 'technical' data bases. The care content related registrations are supplied via one of the previously mentioned registration options (LPZ, Rai-View, ECD/ the organisation's own registration, or the ActiZ registration tool VZ). Data from the clients' consultation is supplied by the measuring agencies on the basis of a format. In the technical data bases data is corrected and transferred to comparable performance scores via 'case mix' for relevant client characteristics (like care intensity). This provides a performance score for each indicator that shows the proportion between the score that can be expected on the basis of the clients' population and the actual score of the organisation.

The comparable performances are fed back within two months to the organisational unit (OE). Firstly this happens in the form of a report for internal use only. This is true for the rough, uncorrected answers to all registration questions and the comparable performance scores for the indicators. Secondly, the comparable performance scores for the indicators are fed back in such a way that they can be delivered by the organisational unit in a user-friendly way to render account (Annual Document, Kiesbeter) and, if applicable for 'Continual Benchmarking' (ActiZ). The organisational unit decides whether or not data will be given to third parties and the organisational unit will always monitor the content of the data that might go to third parties straight from the technical data base.

5. Publication of Performances

Every organisational Unit provides a publication of its performance for the indicators for Responsible care via the Annual Document Social Responsibility (CIBG) and via the Quality Card at www.kiesbeter.nl (RIVM). Besides, performance is registered at an aggregated, anonymous level in an annual, nation-wide report from the Steering Committee.

6. Support

In the course of 2007 support in measuring indicators concerning content of care has been extended further. On the website www.zorgvoorbeter.nl all information, vision documents, instruments and forms are available. There is a Help Desk Responsible Care (responsiblecare@prismant.nl, 030-2345800) and instruction meetings are organised per sector.

II. Set of Indicators Responsible Care

1. Care(treatment)/life plan **29**
1.1 Experiences with care (treatment)/life plan and evaluation 29
1.2 Experienced participation and consultation 30

2. Communication and information **31**
2.1 Experienced treatment 31
2.2 Experienced information 32
2.3 Experienced communication and possibility of reaching staff by phone 33

3. Physical well-being **34**
3.1 Experiences with physical care 34
3.2 Experiences with meals 35

4. Care-related safety **36**
4.1 Decubitus 36
4.2 Nutrition situation 37
4.2a Nutrition situation- weighing 37
4.2b Nutrition situation –inquiry 39
4.3 Fall incidents 41
4.4 Medicine incidents 42
4.5 Psychofarmaca 43
4.6 Degree of vaccination 44
4.6a Degree of vaccination – clients 44
4.6b Degree of vaccination – staff 44
4.7 Incontinence 45
4.7a Incontinence – prevalence 45
4.7b Incontinence – diagnosis 46
4.8 Foley Balloon Catheters 47
4.9 Problem behaviour 48
4.10 Fixation 49
4.11 Policy freedom of movement restricting measures 50
4.12 Experienced professionalism and safety in administering care 51
4.13 Experienced respect of rights concerning restrictions of freedom 53

5. Domestic and living conditions **54**
5.1 Experienced residential comfort 54
5.2 Experienced atmosphere 55
5.3 Experienced privacy (and living space) 56

6. Participation and social handiness **57**
6.1 Experiences with day-time activities and participation 57
6.2 Experienced independence/autonomy 58

7. Mental well-being **59**

7.1 Experiences in the field of mental well-being 59

7.2 Depression 60

8. Safety living/residence **62**

8.1 Experienced safety residential environment 62

8.2 Experienced reliability caregivers 63

8.3 Instruction transfer lifts 64

9. Sufficient and capable staff **65**

9.1 Experienced availability staff 65

9.2 Availability nurse 66

9.3 Availability doctor 67

9.4 Competence reserved treatment 68

10. Coherence in care **69**

10.1 Coherence in care 69

1. Care (treatment) / life plan

1.1 Experiences with care (treatment) / life plan and evaluation

Indicator	**The extent to which clients or representatives experience a good care plan and a good evaluation of that plan**
CQ-index list	Residents<br>Representatives<br>Clients living at home
CQ-index questions	Bewoners<br>27. Have written agreements been drawn up about the care you receive from the nursing home/ care institution? (About what care, which activities, how often, at what times, on which days, et cetera)<br>31. Have you had an evaluation talk with someone from the nursing home/ care institution about whether you are pleased about the care you receive?<br>38. Have you got a regular contact (caregiver/manager) to address in the residence?
	Representatives of residents<br>11. Have written agreements been drawn up about the care the resident receives from the care institution? (What care and what activities, how often, on what days, at what moments, et cetera)<br>15. Have you had an evaluation talk over the past 12 months with someone from the care institution about how things go with care for the resident?<br>22. Have you got a regular contact (caregiver or manager) whom you can address in the institution?
	Clients living at home<br>11. Have written agreements been made with you about the care you receive from the care institution? (What care, as of when, from whom, how often, on what days at what times, et cetera)<br>13. Is your care dossier/file or log book used properly for the exchange of information? (E.g. by your G.P. or your relatives?)<br>17. Have you had an evaluation talk over the past 12 months with someone from the care institution about how you feel about the care given?<br>24. Have you got a regular contact that you can address at the care institution?

1.2 Experienced participation and consultation

Indicator	**The extent to which clients or representatives experience good participation and good consultation.**
CQ-index list	Residents<br>Representatives<br>Clients living at home
CQ-index questions	Residents<br>29. How often do you have a say in matters concerning care or treatment you receive? (The kind of care or support)<br>30. How often do you have a say in matters concerning the times at which and the days on which you receive care or treatment?<br>39. Is the nursing home/ care institution open to your wishes?<br>40. How often does the management or the Board react properly to your questions, suggestions or complaints in your view?<br>43. How often do caregivers discuss with you about what has to be done?
	Representatives of residents<br>13. How often do you participate in the decisions concerning care or treatment that the resident receives (what kind of care/ support)?<br>14. How often do you participate in decisions on the times and days that the resident receives care or treatment?<br>25. Is the care institution open to your suggestions?<br>26. How often does the management or the Board react properly to your questions, suggestions or complaints?<br>30. Do the caregivers and nurses confer with you about what has got to be done?
	Clients living at home<br>14. How often do you participate in decisions about the content of the home care you receive?<br>15. How often do you participate in fixing the times/days on which you receive your home care?<br>16. How often do you participate in decisions about from whom you receive home care (which caregiver)?<br>25. Is the care institution sufficiently open to your suggestions?<br>26. How often does the care institution (the management or the Board) react adequately to your questions, suggestions or complaints?<br>27. How often do your caregivers confer with you about what has got to be done?<br>28. How often do your caregivers ask you if the care they give is up to your standards?

2. Communication and Information

2.1 Experienced treatment

Indicator	**The extent to which clients or representatives experience good treatment.**
CQ-index list	Residents<br>Representatives<br>Clients living at home
CQ-index questions	Residents<br>41. How often do caregivers treat you politely?<br>44. Are the caregivers willing to talk to you about matters that you think did not go well?<br>45. How often do caregivers listen to you attentively?<br>46. How often do caregivers respond to your questions well?
	Representatives of residents<br>28. Are caregivers and nurses willing to discuss with you matters that did not go well in your view?<br>29. Do caregivers and nurses answer your questions well?<br>31. Do caregivers treat residents politely?
	Clients living at home<br>29. How often are caregivers willing to talk to you about matters that did not go well in your opinion?<br>30. How often do caregivers respond to your questions well?<br>47. Do the caregivers treat you in a polite manner?<br>49. Do the caregivers listen to you attentively?

2.2 Experienced information

Indicator	The extent to which clients or representatives experience good information.
CQ-index list	Residents<br>Representatives<br>Clients living at home
CQ-index questions	Residents<br>32. Have you received enough information about what the care institution can offer you? (About residential choice, care, activities, et cetera)<br>33. Have you received enough information about your rights? (E.g. your right to participate in decisions about care and treatment and your right to complain)<br>34. Have you received enough information about what is expected from you? (House rules, your costs/ your own contribution, et cetera)<br>35. Have you received enough information about the care institution's policy on decisions concerning care towards the end of a resident's life?<br>36. Have you received enough information about the clients' council? (About what the council does, how you can reach them, et cetera)<br>37. Do you know to whom you should go with your questions, your problems and your complaints, if any, in the nursing home/care institution?
	Representatives of residents<br>16. Have you received enough information about what your care institution has to offer?<br>17. Have you received enough information about residents' rights? (The right to participate in decisions about care, the right to inspect the care/treatment plan, the right to complain, et cetera)<br>18. Have you received enough information about what is expected from you? (Visiting hours, costs/contributions, et cetera)<br>19. Has the care institution provided you with enough information about its policy concerning decisions about care towards the end of a resident's life?<br>20. Have you received enough information from the care institution about the clients' council? (Function, tasks, how to contact its members, et cetera)<br>21. Do you know to whom you should go with your questions, problems and complaints, if any, in the care institution?
	Clients living at home<br>18. Have you received enough information from the care institution about what it can do for you? (Possibilities in home care, the package of services, et cetera)<br>19 Have you received enough information from your care institution about your rights? (E.g. the right to participate in decisions about care and the right to complain)<br>20. Have you received enough information from the care institution about what is expected from you? (The materials you are supposed to have in the house, your own contribution, et cetera)<br>21. Have you received enough information about the clients' council? (Function, tasks, how to contact its members)<br>22. Are the letters, brochures and flyers understandable to you?<br>23. Do you know to whom you should go in the care institution with your questions, problems and complaints, if any?

2.3 Experienced communication and possibility of reaching staff by phone

Indicator	**The extent to which clients or representatives experience good communication and they can easily reach staff by phone**
CQ-index list	Representatives<br>Clients living at home
CQ-index questions	Client representatives<br>*(The extent to which they experience good communication and the extent to which they can easily reach staff by phone)*<br>23. Can you reach the manager or your contact at the care institution easily by phone?<br>24. If something is the matter with the resident you represent, are you informed quickly enough?<br>27. Have you got good contact with the caregivers?
	Clients living at home<br>*(easy to reach by phone)*<br>31. How often can the care institution easily be contacted by phone during the day from 9 to 5?<br>32. How often can the care institution be easily contacted by phone outside office hours (in the evening, at night, during the weekend)?<br>33. Do you know how and when your contact at the care institution or his/her substitute can be reached by phone?<br>34. If it is impossible for you to reach your contact by phone and you leave a message, will you be called back within one working day?

3. Physical well-being

3.1 Experiences with physical care

Indicator	The extent to which clients or representatives experience good physical care
CQ-index list	Residents<br>Representatives<br>Clients living at home
CQ-index questions	Residents<br>10. Do you receive care at the moments that you want it to be given?<br>11. Is the care given in accordance with what you want?<br>17. Are incontinence products changed in time?
	Clients' representatives<br>34. Is care given at the right moments and as often as is necessary?<br>35. Is care given in a way that befits the client?<br>36. Does the caregiver look clean and well-groomed?<br>39. Are incontinence products changed in time?<br>40. Are the resident's teeth looked after properly?
	Clients living at home<br>56. Do you receive your personal care (like support with taking a shower/ washing, getting dressed, combing your hair, et cetera) at times that you want that care to be given?<br>57. Do you receive care in the way you want to receive it?

3.2 Experiences with meals

Indicator	**The extent to which clients or representatives experience good meals**
CQ-index list	Residents<br>Representatives
CQ-index questions	Residents<br>74. Do the hot meals look well cared- for?<br>75. Are the meals tasty?<br>76. How often do you have a choice of meals?<br>77. Can you choose when to have dinner (around noon or in the evening)?<br>78. Can you choose where you would like to have dinner (in the dining room or your own room/ apartment?
	Clients' representatives<br>67. Do the meals looked well cared-for?<br>68. Is there enough help at dinner time?<br>69. Is there enough time to finish your dinner?<br>70. Are the meals sufficiently spread out over the day? (At 3-hour intervals)

4. Care-related safety

4.1 Decubitus

Indicator	**The percentage of clients with decubitus stage 2 to stage 4 which started in the organisational unit (V&V) during the care period at home (ZT).**
V&V / ZT	Nursing hoe care & Care institution care and Home care
Enumerator	The number of clients with decubitus stage 4 to 4 during the week in which it is measured (started in the organisational unit / during the care period at home).
Denominator	The number of clients whit whom the measurement was carried out on the day / in the week of measuring.
Not to be measured with	• Clients who do not want to be measured for this purpose on the day or in the week that measurements are carried out<br>• Clients with whom it has been put down in writing in the care contract or the care (treatment) / life plan in conference with them that systematic examination for decubitus for the benefit of the Quality Framework will not take place<br>• For Home Care: Clients who have not been indicated for the function nursing and/or personal care (so only those clients should be measured that have been indicated for the function nursing and/or personal care)
Example of Registration Questions	*Is the client suffering from decubitus?*<br>☐ Yes, s/he is.<br>☐ Stage 1<br>☐ Stage 2<br>☐ Stage 3<br>☐ Stage 4<br>☐ No, s/he is not.<br>☐ Unknown:<br>☐ the client does not want to be examined for decubitus<br>☐ unknown for other reasons<br>*If the answer is yes: did decubitus start in the organisational unit / during the care period at home?*<br>☐ Yes<br>☐ No<br>☐ Unknown
Explanation	• It is about decubitus stage 2 to 4 as defined by the CBO http://www.cbo.nl/produkt/richtlijnen/folder20021023121843/decubituskaart.pdf/view). See the instruction material at Zorgvoorbeter.nl for the distinction in stages http://www.zorgvoorbeter.nl/goede-voorbeelden/decubitus/.<br>• In the Testing Framework of November 2005, high-risk patients are mentioned. This is still valid. Whether or not someone is a high-risk client is determined in hind sight with the correction of care dependency. This datum is used in risk correction.

4.2 Nutritional Situation

4.2a Nutritional situation – weighing

Indicator	**The percentage of clients with an unintentional weight loss of more than 3 kilos in the last month or more than 6 kilos over the past 6 months, which is not in line with the agreed treatment policy.**
V&V / ZT	Nursing care & Care Institution care
Enumerator	The number of clients with whom, on the day or in the week of measuring there is an unintentional weight loss of more than 3 kilos over the past month or more than 6 kilos over the past 6 months, which is not in line with the agreed care policy.
Denominator	The number of clients with whom measurements have been carried out on the day/in the week of measuring
Not to be measured with	• Clients who receive terminal care<br>• Clients suffering from cancer<br>• Clients who do not want to or cannot be weighed on the day/in the week of measuring<br>• Clients with whom it has been agreed that there will be no systematic weighing for the benefit of the Quality Framework, as laid down in the care contract or the care (treatment)/ life plan<br>• With V&V: Clients who have not been indicated for the function residence and treatment (so measurements are only carried out for those who have been indicated for both residence and treatment)
Example of Registration Questions	*Has the client, unintentionally, lost more than 3 kilos over the past month or more than 6 kilos over the past 6 months?*<br>☐ Yes, s/he has.<br>☐ This is in line with the agreed treatment policy<br>☐ This is not in line with the agreed treatment policy<br>☐ No, s/he has not.<br>☐ Unknown:<br>☐ The client does not wish to be weighed<br>☐ Unknown for other reasons
Explanation	• This indicator is measured by weighing the client.<br>• If the client indicates that s/he does not want to be weighed, s/he will be asked if oral questions about weight loss may be asked (indicator 4.1b)<br>• Sometimes unintentional weight loss occurs which does fit in with the treatment policy, for example side-effects of a certain type of medication. In such cases the client is not taken up in the enumerator

Read on on page 38.

Page 37 Continued.

Explanation	References:<br>- Kruizenga, H.M., J.C. Seidellb, H.C.W. de Vetc, N.J. Wierdsmaa, M.A.E. van Bokhorst-de van der Schueren, 2005. *Development and validation of a hospital screening tool for malnutrition: the short nutritional assessment questionnaire (SNAQ).* Clinical Nutrition 2005, 24, 75-82<br>- Kruizenga, H.M., Maurtis W van Tulder, Jaap C Seidell, Abel Thijs, Herman J Ader and Marian AE Van Bokhorst-de van der Schueren, 2005. *Effectiveness and cost-effectiveness of early screening and treatment of malnourished patients*.<br>The American Journal of Clinical Nutrition, 2005, 82, 1082-9<br>- http://www.snellerbeter.nl/fileadmin/snellerbeter/documenten/Indicatoren/Basisset_Prestatie-indicatoren_2007.pdf<br>- Elia, M., L Zellipour, RJ Strattton. 2005. *To Screen or not to screen for adult malnutrition?* Clinical Nutrition, 2005, 24, 867-884<br>http://www.snellerbeter.nl/programmasb1/ondervoeding/<br>Project ‘Early recognition and treatment of malnutrition in Dutch hospitals’

4.2b Nutrition situation - Inquiry

Indicator	**Percentage of clients with unintentional weight loss of more than 3 kilos over the previous month or more than 6 kilos over the previous 6 months**
V&V / ZT	Nursing Care & Care Institution Care and Home Care
Enumerator	The number of clients who, on the day/in the week of measuring, had an unintentional weight loss of more than 3 kilos over the previous month or more than 6 kilos over the previous 6 months.
Denominator	The number of clients with whom measurements have been carried out on the day/in the week of measuring
Not to be measured with	• Clients who receive terminal care or are suffering from cancer<br>• Clients whose unintentional weight loss has already been established by weighing (indicator 4.1a)<br>• Clients who, on the day / in the week of measuring, could not or would not be asked about this<br>• Clients with whom has been agreed in the care (treatment) / life plan that systematic examination concerning weight loss for the benefit of the Quality Framework will not be carried out
Example of Registration Questions	*Have you lost weight unintentionally?*<br>☐ Yes, I have<br>☐ More than 6 kilos over the past 6 months or more than 3 kilos over the past 4 weeks? ----> 1 point<br>☐ Less than mentioned above<br>☐ No, I have not.<br>☐ Unknown:<br>☐ The client wishes not to be weighed<br>☐ unknown for other reasons<br>*Have you had less of an appetite over the past 4 weeks?*<br>☐ Yes, I have. ----> 1 point<br>☐ No, I have not.<br>☐ No, I have not.<br>☐ The client wishes not to be weighed<br>☐ Unknown for other reasons<br>*Have you used liquid food or have you been drip-fed?*<br>☐ Yes, I have. ----> 1 point<br>☐ No, I have not.<br>☐ Unknown:<br>☐ The client wishes not to be weighed<br>☐ Unknown for other reasons<br>*Scores of client: 0/1/2/3*

Read on on page 40.

Page 40 Continued.

Explanation	• This indicator is measured by inquiring with the client<br>• Together the questions form the SNAQ (Short Nutritional Assessment Questionnaire).<br>• References: see indicator 4.2a
Remarks	considering the enumerator: the number of clients with a score of 2 or more on the chosen nutritional situation scale; the number of clients measured on the day/in the week of measuring

4.3 Fall incidents

Indicator	**The percentage of clients that has been involved in a fall incident over the last 30 days**
V&V / ZT	Nursing care & Care Institution Care and Home Care
Enumerator	The number of clients involved in falling incidents over the past 30 days
Denominator	The number of clients with whom measurements were carried out in the week/ on the day of measuring
Not to be measured with	not applicable
Example of Registration Questions	*Has the client been involved in a fall incident over the past 30 days?*<br>☐ Yes, s/he has.<br>☐ No, s/he has not.<br>☐ Unknown
Explanation	• In answering this question preferably various sources are used simultaneously: the client's dossier, the caregiver's and the client's memory and the MIC registration<br>• With the term 'fall' we mean: suddenly and unintentionally hitting the ground from a vertical or horizontal position. Whether an incident is a fall or not is therefore not based on the fact that the patient got hurt.<br>• Background information: see http://www.zorgvoorbeter.nl/goede-voorbeelden/valpreventie/

4.4 Medicine incidents

Indicator	**The percentage of clients that has been involved in a medicine-related incidents over the past 30 days**
V&V / ZT	Nursing Care & Care Institution Care
Enumerator	The number of clients that has been involved in a medicine-related incident
Denominator	The number of clients with whom measurements have been carried out on the day/in the week of measuring
Not to be measured with	Clients who are in full control of their medicines
Example of Registration Questions	*Has the client been involved in a medicine-related incident over the past 30 days?*<br>☐ Yes, s/he has.<br>☐ The medicine was not offered to the client<br>☐ The wrong dose was handed to the client<br>☐ The medicine was given at the wrong time<br>☐ The client did not take the medicine<br>☐ The wrong kind of medicine was given<br>☐ Other<br>☐ No, s/he has not.<br>☐ Unknown:<br>☐ The client is in full control of her/his medicines<br>☐ Unknown for other reasons
Explanation	• In answering this question preferably various sources are used simultaneously: the client's dossier, the caregiver's and the client's memory and the MIC registration.<br>• With the term 'medicine-related incident' we mean one or more of the situations mentioned under registration question.<br>• Background information: http://www.zorgvoorbeter.nl/goede-voorbeelden/medicatieveiligheid/-Guideline Medication safety ActiZ

4.5 Psychofarmaca

Indicator	**a) The percentage of clients that has used anti-psychotic, anti-anxiety medication or hypnotica one or more days over the past week**<br>**b) The percentage of clients that has used anti-depressants one or more days over the past week**
V&V / ZT	Nursing Home Care & Care Institution Care
Enumerator	a) the number of clients that has used anti-psychotic, anti-anxiety medication or hypnotica one or more days over the past week<br>b) the number of clients that has used ant-depressants one or more days over the past week
Denominator	The number of clients with whom measurements were carried out on the day/in the week of measuring
Not to be measured with	Clients who are in full control of their medication
Example of Registration Questions	*How many days over the past week has the client received the following medicines?*<br>Way of scoring: fill in 0 for none; fill in the number of days if the medication is effective for more than one day (with a maximum of 7 days)<br>☐ Anti-psychotics ... days<br>☐ Anxiolytica ... days<br>☐ Hypnotica ... days<br>☐ Anti-depressants ... days<br><br>☐ Unknown:<br>☐ The client is in full control of medication<br>☐ Unknown for other reasons
Explanation	• The use of anti-depressants will be considered in relation to prevalence of depression among clients.<br>• If the people who carry out measurements are not sufficiently familiar with the names of substances in medicines, it is recommended that things are arranged with the dispensing chemist in such a way that lists with brand names and names of substances are provided.<br>• Background:<br>- http://www.zorgvoorbeter.nl/goede-voorbeelden/ medicatieveiligheid/<br>- Minimum Data Set Resident Assessment Instrument (RAI) , intramural and extramural version
Remarks	In future enumerator and denominator must be under indicator b): enumerator: the number of clients that has used anti-depressants; denominator: the number of clients that scored 3 points on the depression scale (indicator 7.2)

4.6 Degree of vaccination

4.6a Degree of vaccination - Clients
4.6b Degree of vaccination - Staff

Indicator	**a) the percentage of clients and b) the percentage of staff that was vaccinated against influenza between the second week of September and the second week of November**
V&V / ZT	Nursing care& Care Institution care
Enumerator	a) The number of clients that was vaccinated against influenza between the second week of September and the second week of November last year<br>b) The number of staff that was vaccinated against influenza between the first week of September and the second week of November last year
Denominator	a) The number of clients that was measured during the week / on the day of measuring<br>b) The number of staff that was employed at the end of the first week of November
Not to be measured with	not applicable
Example of Registration Questions	a)<br>*Was the client vaccinated against influenza between the second week of September and the second week of November last year?*<br>☐ Yes, s/he was.<br>☐ No, s/he was not.<br>☐ The client did not want to be vaccinated.<br>☐ For other reasons<br>☐ Unknown<br>b)<br>• *How many staff (permanent or temporary) did the organisational unit have in the period between the second week of September and the second week of November?*<br>• *How many staff were vaccinated against influenza in the period between the second week of September and the second week of November?*
Explanation	• For the definition of influenza: see guideline prevention of influenza of the Dutch Association of Nursing Home Doctors<br>• Also see RIVM, 2002, http://www.rivm.nl

4.7 Incontinence

4.7a Incontinence – prevalence

Indicator	**Percentage of clients that has urinary incontinence a couple of times per week or every day**
V&V / ZT	Nursing Care & Care Institution Care and Home Care
Enumerator	The number of clients that has urinary incontinence a couple of times per week or every day (including the clients who have catheters)
Denominator	The number of clients with whom measurements have been carried out on the day / in the week of measuring
Not to be measured with	• clients who did not want to or could not be tested for incontinence on the day / in the week of measuring<br>• clients with whom it was agreed and put down in writing in the care contract or in the care (treatment) / life plan that systematic testing for urinary incontinence is not to be carried out<br>• Home care: clients who have not been indicated for the function nursing and/or personal care (so only if the client has been indicated for the function nursing and/or personal care)
Example of Registration Questions	*How often is the client incontinent?*<br>☐ The client has a catheter<br>☐ Never<br>☐ Three or four times a month<br>☐ A couple of times per week<br>☐ Every day<br>☐ Unknown:<br>☐ The client wishes not to be tested for urinary incontinence<br>☐ Unknown for other reasons
Explanation	• The term urinary incontinence means: any form of involuntary loss of urine. Urine retention, therefore, is not regarded as incontinence<br>• If a client has a catheter, this should be indicated with the registration question. These clients are regarded as incontinent for this indicator and so they count for the enumerator.<br>• This indicator is in line with the National Prevalence Measuring of Care Problems (LPZ) University of Maastricht

4.7b Incontinence - diagnosis

Indicator	**The percentage of clients with incontinence on the day of/in the week of measuring in whose diagnosis a doctor or incontinence nurse was involved**
V&V / ZT	Nursing Home Care & Care Institution Care and Home Care
Enumerator	The number of clients with incontinence on the day /in the week of measuring (see 4.7a)
Denominator	
Not to be measured with	• Clients who are not incontinent (every day or a couple of of times a week) (so clients should only be measured if they are incontinent)<br>• Clients who do not wish to be or cannot be examined for urinary inconti-nence on the day/in the week of measuring<br>• Clients with whom it has been agreed (and put down in writing) in the care contract or the care (treatment)/ life plan that systematic testing for urinary incontinence is not to be carried out for the benefit of the Quality Framework<br>• Home Care: Clients who have not been indicated for the function nursing and / or personal care (so clients should only be measured if they have been indicated for the function nursing and/ or personal care)
Example of Registration Questions	*Who has diagnosed urinary incontinence?*<br>☐ G.P.<br>☐ Nursing Home doctor<br>☐ Urologist<br>☐ Incontinence nurse<br>☐ Not diagnosed<br>☐ Unknown
Explanation	• The term urinary incontinence means: any form of involuntary loss of urine. Urine retention is not regarded as incontinence.<br>• Clients with a catheter are regarded as incontinent for this indicator.<br>• This indicator is in line with the National Prevalence Measurement Care Problems (LPZ) University of Maastricht

4.8 Foley Balloon Catheter

Indicator	**The percentage of clients with a Foley catheter that was inserted more than 2 weeks ago**
V&V / ZT	Nursing Home Care & Institution Care and Home Care
Enumerator	The number of clients that has had a catheter for more than 2 weeks on the day/in the week of measuring
Denominator	The number of clients with whom measurements were carried out on the day/in the week of measuring
Not to be measured with	• Home Care: Clients who have not been indicated for the function nursing and / or personal care (so clients should only be measured if they have been indicated for the function nursing and/ or personal care)
Example of Registration Questions	*Has the client got a Foley Balloon catheter?*<br>☐ Yes, s/he has:<br>☐ The catheter has been inserted within the organisational unit<br>☐ The catheter has not been inserted within the organisational unit<br>☐ The catheter was inserted more than 2 weeks ago<br>☐ The catheter was inserted 14 days ago or earlier<br>☐ No, s/he has not.
Explanation	• With the registration question it has to be indicated whether or not the catheter has been inserted within the organisational unit. Therefore, clients who already had the catheter inserted when they started to receive care will be left out of account.<br>• This indicator is in line with the National Prevalence Measurement Care Problems (LPZ) University of Maastricht

4.9 Problem behaviour

Indicator	**The percentage of clients that has shown problem behaviour towards staff and/or other clients**
V&V / ZT	Nursing Home Care & Care Institution Care
Enumerator	The number of clients that has shown one or more symptoms of problem behaviour towards staff and/or other clients over the past 7 days
Denominator	The number of clients with whom measurements were carried out on the day/in the week of measuring
Not to be measured with	• Clients who wish not to be or cannot be tested for problem behaviour on the day/in the week of measuring<br>• Clients with whom it has been agreed (and put down in writing) in the care contract or the care (treatment)/ life plan that systematic testing for problem behaviour is not to be carried out for the benefit of the Quality Framework
Example of Registration Questions	*Has the client shown the following behavioural symptoms over the past 7 days?*<br>☐ Yes, s/he has, namely:<br>☐ Verbal problem behaviour, description: the client shows threatening behaviour towards others, shouts at them and uses swear words<br>☐ Physical problem behaviour, description: the client hits, pushes, scratches or (sexually) intimidates others<br>☐ Anti-social or disruptive behaviour, description: the client makes upsetting noises, is loud, shouts, abuses him/herself, shows sexual or exhibitionistic behaviour, covers him/herself or throws food or faeces, hoards or rummages in other people's belongings<br>☐ Refusing to accept care, description: the client refuses to take medication or accept injections, refuse to accept help at meal times or with day-to-day activities<br>☐ No, s/he has not.<br>☐ Unknown:<br>☐ The client wishes not to be tested for problem behaviour<br>☐ Unknown for other reasons
Explanation	This indicator is in line with the Minimum Data Set Resident Assessment Instrument (RAI), version intramural and extramural

4.10 Fixation

Indicator	**The percentage of clients to whom fixation was applied**
V&V / ZT	Nursing Home Care & Care Institution Care
Enumerator	The number of clients to whom fixation has been applied over the past 7 days by means of a restraint (for example the Swedish belt) or by having them chair-bound or by means of a restraining table-top.
Denominator	The number of clients with whom measurements were carried out on the day/in the week of measuring
Not to be measured with	-
Example of Registration Questions	*Has fixation been applied to the client overt the past 7 days by means means of a restraint (Swedish belt), by means of having the client chair-bound or by means of a restraining table-top?*<br>☐ Yes, it has.<br>☐ No, it has not.<br>☐ Unknown
Explanation	This indicator is in line with the IGZ Forms 2003-2006

4.11 Policy Fixation Measures

Indicator	**The organisational unit can prove that they have a demonstrable policy for the prevention of restricting measures concerning freedom of movement**
V&V / ZT	Home Care
Example of Registration Questions	*Can you prove that you have a policy concerning the prevention of restricting measures related to freedom of movement?*<br>☐ Yes, we can.<br>☐ No, we cannot.
Explanation	• The organisational unit should be able to demonstrate this through its own registration<br>• This indicator is in line with the IGZ Forms 2003-2006a

4.12 Experienced professionalism and safety in administering care

Indicator	**The extent to which clients or representatives experience adequate professionalism (and safety) in administering care**
CQ-index list	Residents<br>Representatives<br>Clients living at home
CQ-index questions	Residents<br>18. Do caregivers and nurses work professionally?<br>19. Are nursing activities carried out professionally? (Like injections, taking care of wounds and stomas)<br>20. Do the various caregivers attune their care and treatment to one another properly?<br>22. Are your caregivers well-informed about your illness (-es) or health problems?<br>23. Do caregivers and nurses take into consideration whether or not you can do certain tasks yourself?<br>24. Do caregivers take your complaints concerning your health seriously? (Caregivers, nurses, doctor, physiotherapist, et cetera)<br>26. Do you receive adequate information about medication if you are given medicine that you have not used earlier? (Name, effects, potential side-effects)<br>28. Does the nursing home / care institution observe the agreements about the care and treatment you receive properly?
	Representatives of residents<br>12. Does the care institution observe the agreements about care and treatment of the resident?<br>42. Do caregivers and nurses do a professional job?<br>43. Are nursing activities (e.g. injecting, taking care of wounds, stomas) done professionally?<br>44. Do the various caregivers (nurses, caregivers, doctors, physiotherapists, et cetera) attune their care for the client to one another well?<br>46. Are the caregivers and nurses well able to deal with the clients?<br>47. Are the caregivers well-informed about the resident's health problems (caregivers, nurses, doctors, physiotherapists, et cetera)?<br>48. Do the caregivers and nurses sufficiently take into consideration what the resident can or cannot do him/herself?<br>49. Are complaints about health problems taken seriously by caregivers, nurses, doctors, physiotherapists, et cetera?<br>51. Do you get adequate information about medicines (name, effect, potential side-effects) if the resident receives a new medicine for the first time?

Read on page. 52.

Page 51 Continued.

CQ-index questions	Clients living at home<br>12. Does the care institution observe the agreements related to care?<br>37. Do the various caregivers coordinate the home care you receive well?<br>41. Do the caregivers work professionally?<br>42. Do the caregivers work independently? (Do they know what should be done?)<br>43. Do the caregivers do the cleaning adequately?<br>44. Are the caregivers competent in carrying out nursing activities (injecting, taking care of wounds and stomas)?<br>45. Are caregivers well-informed about your illness(-es) or health problems?<br>50. Do the caregivers work in the way that you want them to work?<br>58. Do caregivers take into consideration whether or not you can do certain tasks yourself?<br>59. Do your caregivers pay attention to changes in your health situation or do they tell you that they notice such changes?<br>60. Do your caregivers pay attention to your correct use of medicines? (That you take the prescribed medicine at the correct time in the correct way)<br>61. Are your complaints about your health taken seriously by your caregivers?

4.13 Experienced respecting of rights concerning restriction of freedom of movement

Indicator	**The extent to which representatives experience adequate respect for clients' rights in relation to restriction of freedom of movement**
CQ-index list	Representatives
CQ-index questions	Representatives of residents<br>52. Have the resident's rights concerning the application of measures concerning restriction of freedom of movement been discussed with you? (Restraints, high bed-railings, table-top, being chair-bound)<br>53. Is permission asked from you about application of measures restricting freedom of movement (like restraints, high bed-railings, table-top, being chair- bound)?

5. Domestic and living conditions

5.1 Experienced living comfort

Indicator	**The extent to which clients or representatives experience adequate living comfort**
CQ-index list	Residents<br>Representatives
CQ-index questions	Residents<br>51. Is the temperature in your home comfortable?<br>52. Is your living area cleaned properly (living room, bedroom, toilet and shower/bathroom)?
	Representatives of residents<br>54. Is the temperature in the living area comfortable?<br>55. Is the living area (living room, bed-room, toilet and shower/bathroom) cleaned properly?

5.2 Experienced atmosphere

Indicator	The extent to which clients or representatives experience a good atmosphere
CQ-index list	Residents<br>Representatives
CQ-index questions	Residents<br>53. Are the public rooms like living room, corridor and Lobby pleasant places to be? (Comfortable, clean, nice smell)<br>54. Do the residents interact pleasantly and sociably?<br>72. Does the home offer enough opportunities for clients to have contact with fellow-residents, locals, et cetera?<br>79. Is there a pleasant atmosphere at meal times?
	Representatives of residents<br>56. Are public rooms like living room, corridor and lobby pleasant places to be? (Comfortable, clean, nice smell)<br>58. Do the residents interact pleasantly and sociably?<br>65. Does the home offer enough opportunities for clients to have contact with others?

5.3 Experienced privacy (and living accommodation)

Indicator	**The extent to which clients or representatives experience adequate privacy (and living accommodation)**
CQ-index list	Residents<br>Representatives<br>Clients living at home
CQ-index questions	Residents<br>*(Privacy and living accommodation)*<br>15. Do you have enough privacy while using the toilet?<br>56. Have you got enough room to live in?<br>57. Can you decide for yourself how your living area is furnished? (With your own furniture, belongings, etc.) (Please note: this question also applies to shared accommodation)<br>58. Can you and your visitors withdraw to a quiet place?<br>59. Have you got enough moments to be on your own?<br>60. Do caregivers and staff leave you alone if you want them to?
	Residents' representatives<br>*(Privacy and accommodation)*<br>59. Does the resident have enough room to live in?<br>60. Can the resident withdraw to a quiet place with visitors?<br>61. Does the care institution enable the resident to furnish his/her own room or living area? (With their own belongings, et cetera)
	Clients living at home<br>*(Privacy)*<br>68. Is home care an infringement of your daily life because of the caregivers who come into your home (your privacy, the daily routine and activities)?

6. Participation and social handiness

6.1 Experiences with how clients spend their days and with participation

Indicator	**The extent to which clients or representatives experience adequate possibilities to spend the day and to participate in society.**
CQ-index list	Residents<br>Representatives<br>Clients living at home
CQ-index questions	Residents<br>68. Have you got enough possibilities to participate in activities and daily matters?<br>69. Are you well-informed about activities in the home?<br>70. Does the home organise enough activities?<br>71. Do you enjoy the activities that are organised?<br>73. Is there support from caregivers, volunteers or relatives if you want to go somewhere? (Outside, visiting others, outings, activities, etc.)
	Residents' representatives<br>62. Has the resident enough opportunities to participate in activities and daily matters?<br>63. Does the home organise enough activities?<br>64. Are the activities that are organised enjoyed by the residents?<br>66. Is there support for the residents when they want to go outside or on an outing? (Help from caregivers, volunteers, relatives, etc.)
	Clients living at home<br>77. Is there any support from caregivers, volunteers or relatives if you want to go somewhere? (Outside, visiting others, outings, activities, etc.)<br>78. Does your caregiver offer you enough support in finding ways to spend the day, social contacts and activities?<br>79. Does your caregiver offer you enough practical support in arranging practical matters as making phone calls, filling in forms, arranging aids or financial matters, etc.)

6.2 Experienced independence / autonomy

Indicator	**The extent to which clients or representatives experience adequate independence / autonomy**
CQ-index list	Residents<br>Representatives<br>Clients living at home
CQ-index questions	Residents<br>12. Can you go to bed and get up when you want to?<br>61. Do you decide how you want to spend the day?<br>62. Can you go any where you like in the nursing home /care institution?<br>63. Can you go anywhere you like outside the home?
	Residents' representatives<br>37. Can the resident go to bed and get up at times that befit the resident?
	Clients living at home<br>69. Can you manage daily life easily with home care?<br>70. Can you decide how you want to spend the day?<br>71 Can you go to bed and get up when you want to?<br>72. Are the times at which you receive home care convenient to you?<br>74. Can you the things that matter to you?<br>75. Can you go anywhere you like in the home?<br>76. Can you go anywhere you like outside the home?

7. Mental well-being

7.1 Experiences concerning mental well-being

Indicator	**The extent to which clients or representatives experience adequate mental support**
CQ-index list	Residents<br>Representatives<br>Clients living at home
CQ-index questions	Residents<br>47. Do caregivers and nurses pay enough attention to how you feel?<br>48. How often do you worry about things?<br>49. Do you feel lonely?<br>50. Is there a spiritual counsellor available for you in the home? (E.g. a minister/priest, humanistic counsellor or social worker)<br>55. Do you feel at home here?
	Residents' representatives<br>33. Does staff pay enough attention to how the resident feels?
	Clients living at home<br>66. Do you feel at home in your own apartment?<br>80. How often do you worry about things?<br>81. Do you feel lonely?<br>82. Does your caregiver pay sufficient attention to how you are doing?<br>83. Does your caregiver offer enough emotional support in conversations and is s/he a good listener?

7.2 Depression

Indicator	**The percentage of clients that have shown signs of depression over the past three days**	
V&V / ZT	Nursing Home Care & Care Institution Care and Home Care	
Enumerator	The number of clients that scored 3 or more points on the depression scale on the day/in the week of measuring	
Denominator	The number of clients with whom measurements have been carried out on the day/in the week of measuring	
Not to be measured with	• Clients who, on the day/in the week of measuring, cannot or do not want to be tested for depression<br>• Clients with whom it has been agreed (and put down in writing) in the care contract or the care (treatment)/ life plan that systematic testing for depression is not to be carried out for the benefit of the Quality Framework<br>• Home Care: Clients who have not been indicated for the function nursing and / or personal care (so clients should only be measured if they have been indicated for the function nursing and/ or personal care)	
Example Registration Questions	*How often has the client shown these signs (Home care: during the last three contact moments) over the past three days?*<br><br>Scoring:<br>0: if there is no sign of depression<br>1: if the signs have been shown on one or two of the pasty three days (ZT: three contact moments)<br>2: if the signs have been shown daily (ZT: every time) over the past three days (ZT: three contact moments)	
	Signs	Score (0, 1, 2)
	☐ Negative expressions like, " I don't care any longer; I wish I were dead; what's the use; I regret having lived for so long; let me die."	
	☐ Continually being angry with oneself or others; e.g. is easily annoyed, angry with having to stay in the home or angry with the care received	

Read on page 61.

Page 60 Continued.

	☐ Expressions (verbal and non-verbal) of anxiety that do not seem realistic, like the fear to be left alone; to be alone; to be together with others; an intense fear of specific objects or situations	
	☐ Continuous complaining about ones health, like constantly asking to see a doctor; being obsessively anxious about bodily functions	
	☐ Repeated fearful complaining/anxiety not related to health, like constant seeking attention or reassurance about daily life, meals, laundry, clothe, interaction with others	
	☐ Sad, hurt facial expression with deep wrinkles; knitting one's brow all the time	
	☐ Crying, bursting into tears	
	Total score	
	Is the score higher than three?<br>☐ Yes, it is.<br>☐ No, it is not.<br>☐ Unknown:<br>☐ The client wishes not to be tested for depression<br>☐ Unknown for other reasons	
Explanation	This indicator is in line with the Minimum Data Set Resident Assessment Instrument (RAI), version intramural and extramural	

8. Safety Living /Residence

8.1 Experienced safety domestic / living area

Indicator	**The extent to which clients or representatives experience a safe living environment**
CQ-index list	Residents<br>Representatives<br>Clients living at home
CQ-index questions	Residents<br>64. Do you feel safe at home?<br>66. Are you worried about theft?<br>67. Do you know what to do in case of fire?
	Residents' representatives<br>57. Is there enough supervision (staff that is on the alert for potentially unsafe situations) in the living room, corridors, lifts, etc.)?
	Clients living at home<br>62. Do caregivers pay enough attention to your safety and the prevention of accidents in and around the home? (E.g. by watching out for loose mats/rugs on slippery floors)<br>63. Do your caregivers point out to you the possibilities of adapting your home or the existence of certain aids?<br>64. Do your caregivers pay attention to the sell-by date of food? (to prevent food poisoning)<br>65. Has the care institution discussed with you what you should do in case of emergency? (Which tel. number you can dial, when and how you can raise the alarm, etc.)

8.2 Experienced reliability of staff

Indicator	**The extent to which clients or representatives experience staff to be adequately reliable**
CQ-index list	Representatives<br>Clients living at home
CQ-index questions	Residents' representatives<br>41. Do caregivers treat residents' belongings with great care? (Glasses, jewellery, clothing, hearing aids, dentures, etc.)
	Clients living at home<br>51. Do caregivers stick to agreed tasks? (Does the work get done?)<br>52. Do caregivers stick to the appointed times? (do they come on time, don't they leave early?)<br>53. Do caregivers deal with your confidential personal data and private matters respectfully?<br>54. Do caregivers respectfully treat your belongings (furniture, crockery, clothing, etc.)?<br>55. Can you fully trust your caregivers?<br>67. Do you feel safe and at ease when caregivers are with you?

8.3 Instruction transfer lifts

Indicator	**The organisational unit can prove that staff members working with transfer 1lifts have been instructed to do this.**
V&V / ZT	Nursing Home Care and Care Institution Care and Home Care
Example Registration Questions	*Can you prove that staff members working with transfer lifts have been instructed to do this?*<br>☐ Yes, we can:<br>☐ The last time instruction was given was ... (year, month)<br>☐ No, we cannot.<br>☐ Not applicable, we do not work with transfer lifts.
Explanation	• The organisational unit should be able to show in writing that this particular training has taken place<br>• The reason for the organisational unit to focus on one specific aid (transfer lift) is that this will be exemplary for how an organisational unit deals with instructions and safety in relation to other materials and aids.
	• This indicator is in line with IGZ Forms 2003-2006

9. Sufficient and competent staff

9.1 Experienced availability of staff

Indicator	**The extent to which clients or representatives experience sufficient availability of staff (and continuity)**
CQ-index list	Residents<br>Representatives<br>Clients living at home
CQ-index questions	Residents<br>*(Availability staff)*<br>14. Do you get help in time when you have to go to the toilet?<br>21. Is there enough staff in the home?<br>25. Do you get help quickly when you are ill or in pain?<br>42. Do caregivers spend enough time on you?<br>65. Does the caregiver react within 5 minutes after you have called him/her?
	Residents' representatives<br>*(Availability of staff)*<br>32. Do caregivers spend enough time on the resident?<br>38. Does the resident get help in time when s/he has to go to the toilet?<br>45. Is there enough staff in the home?<br>50. Does the resident get help quickly when s/he is ill or in pain?
	Clients living at home<br>*(Availability staff and continuity)*<br>35. How many different caregivers come into your apartment in one month? (Under normal conditions, outside the holiday period)<br>36. What do you think about the number of different caregivers that come to help you?<br>38. Are you informed in time about caregivers coming at other times than usual or if a caregiver is ill or on holiday?<br>39. Are substitutes arranged well if your regular caregiver is ill or has a day off?<br>40. Are the substitute caregivers well-informed about the tasks/chores to be carried out?<br>48. Do caregivers spend enough time on you?<br>73. Is home care flexible in adapting the moments of care giving to your wishes? (Different time, another day)

9.2 Availability nurse

Indicator	**The organisational unit can show that for the function Residence combine with Nursing and/or Treatment there is a nurse available seven times 24 hours and that the nurse can be on location within 10 minutes**
V&V / ZT	Nursing Home Care and Care Institution Care
Example Registration Questions	*Can you show that for the function Residence combined with Nursing and/or Treatment there is a nurse available seven times 24 hours and that the nurse can be on location within 10 minutes?*<br>☐ Yes, we can.<br>☐ No, we cannot.<br>☐ But we have a small-scale residence in which contact by telephone with a nurse within 10 minutes can be arranged<br>☐ We cannot show this
Explanation	• The organisational unit should be able to show this through its own registration<br>• For small-scale residences and in the case of light care intensity it is conside-red to be acceptable if a nurse can be contacted within 10 minutes. This deviation of the rule, if it occurs, is explained with motivation.<br>• This indicator is the result of consensus among the field parties of the Steering Committee Responsible Care VV&T

9.3 Availability doctor

Indicator	**The organisational unit can show that for the function Residence combined with Nursing and/or Treatment a doctor can be reached and called in seven times 24 hours and that the doctor will react within 10 minutes and be on location within 30 minutes?**
V&V / ZT	Nursing Home Care & Care Institution Care
Example Registration Questions	*Can you show that for the function Residence combined with Nursing and/or Treatment a doctor can be reached seven times 24 hours who can be on location within 30 minutes?*<br>☐ Yes, we can<br>☐ No, we cannot
Explanation	• The organisational unit should be able to show this through its own registration<br>• in a nursing home the doctor is a nursing home doctor<br>• in a care institution the doctor is a qualified doctor<br>• at a nursing unit in a care institution the qualified doctor is supported by a nursing home doctor via an ‘ on call’ construction<br>• this indicator is the result of consensus among the field parties of the Steering Committee Responsible care VV&T

9.4 Competence reserved treatment

Indicator	**The organisational unit can show that over the reported past year competencies of staff that carry out reserved or risky treatment have been tested (practical test) and found to be up to standard**
V&V / ZT	Nursing Home Care & Care Institution Care and Home care
Example Registration Questions	*Can you prove that by means of a 'practical test' it has been tested if competencies of staff carrying out reserved or risky treatment are up to standard?*<br>☐ Yes, we can.<br>☐ The most recent practical test was carried out in … - …. (month, year)<br>☐ No, we cannot.
Explanation	• Reserved treatment encompasses actions that may be carried out by non-doctors, only when instructed to do so by a doctor. These forms of treatment have been laid down the Law BIG (Chapter IV, clause 35 – 39). Risky treatment encompasses actions, for example with technical aids that require specific expertise.<br>• In a 'practical test' the reserved treatment is carried out in the presence of an expert. This can be a doctor, a specialised nurse or a practical supervisor. The expert decides whether the treatment was done properly and reports it. It is essential that you should be able to show by means of a document (report, registration, etc.) that staff competency has been tested in such a manner.<br>• This indicator is in line with IGZ Forms 2003-2006

10. Coherence in care

10.1 Coherence in care

Indicator	**The extent to which clients or representatives experience adequate coherence in care**
CQ-index list	Clients living at home
CQ-index questions	Clients living at home<br>46. Do caregivers cooperate well with other disciplines like the G.P., specialists, physiotherapists, dieticians?

Appendix 3 Survey Indicators for Responsible care VVT

Themes and indicators	Client-bound indicators			Indicators concerning content of care			
	Client consultation CQ-Index			Self-monitoring organisation organisational level		Self-monitoring organisation clients' level	
	VV	PG	ZT	VV	ZT	VV	ZT
**1. Care (treatment)/life plan**							
1.1 Experiences with care (treatment) / life plan and evaluation	VV	PG	ZT				
1.2 Experienced participation	VV	PG	ZT				
**2. Communication and information**							
2.1 Experienced treatment	VV	PG	ZT				
2.2 Experienced information	VV	PG	ZT				
2.3. Experienced communication and possibility to reach staff by phone	-	PG	ZT				
**3. Physical well-being**							
3.1. Experiences with physical care	VV	PG	ZT				
3.2. Experiences with meals	VV	PG	-				
**4. Care-related safety**							
4.1. Decubitus						VV	ZT
4.2. Nutrition situation							
4.2a Nutrition situation - weighing						VV	-
4.2b Nutrition situation - inquiry						VV	ZT
4.3. Fall incidents						VV	ZT
4.4. Medicine incidents						VV	-
4.5. Psychofarmaca						VV	-
4.6. Degree of vaccination							
4.6a Degree of vaccination - clients						VV	-
4.6b Degree of vaccination - staff				VV	-		
4.7. Incontinence							
4.7a Incontinence - prevalence						VV	ZT
4.7b Incontinence - diagnosis						VV	ZT
4.8. Foley Balloon Catheters						VV	ZT
4.9. Problem behaviour						VV	-
4.10. Fixation						VV	-
4.11. Policy measures restricting freedom of movement				-	ZT		
4.12. Experienced professionalism and safety in care	VV	PG	ZT				
4.13. Experienced respect for rights concerning measures restricting freedom of movement	-	PG	-				

Client-bound indicators

Indicators concerning content of care

Themes and indicators	Client-bound indicators			Indicators concerning content of care			
	Client consultation CQ-Index			Self-monitoring organisation organisational level		Self-monitoring organisation clients' level	
	**VV**	**PG**	**ZT**	**VV**	**ZT**	**VV**	**ZT**
**5. Domestic and living conditions**							
5.1. Experienced living comfort	VV	PG	-				
5.2. Experienced atmosphere	VV	PG	-				
5.3. Experienced privacy (and living area)	VV	PG	ZT				
**6. Participation and social handiness**							
6.1. Experiences with how clients spend their days and with participation	VV	PG	ZT				
6.2. Experienced independence /autonomy	VV	PG	ZT				
**7. Mental well-being**							
7.1. Experiences concerning mental well-being	VV	PG	ZT				
7.2. Depression						VV	ZT
**8. Safety living/residence**							
8.1. Experienced safety domestic / living area	VV	PG	ZT				
8.2. Experienced reliability caregivers	-	PG	ZT				
8.3. Instruction transfer lifts				VV	ZT		
**9. Sufficient and competent staff**							
9.1. Experienced availability of staff	VV	PG	ZT				
9.2. Availability nurse				VV	-		
9.3. Availability doctor				VV	-		
9.4. Competence reserved treatment				VV	ZT		
**10. Coherence in care**							
10.1. Experiences with chain care	-	-	ZT				
**Number of indicators**	**15**	**18**	**15**	**5**	**3**	**13**	**7**

VV= (Verpleging en Verzorging) Nursing and Care, PG=(Vertegenwoordigers van Psychogeriatrische bewoners) Representatives of psycho-geriatric residents, ZT= (Zorg Thuis) Home care

Colophon

Text
Stuurgroep Kwaliteitskader Verantwoorde Zorg
(Steering committee Responsible Care)

Translation
Mitchell Alexander
Crossroads Language Service

Design
Pier 19, Utrecht

Print
We Print Together (WPT), Rijswijk

May 2008, The Netherlands

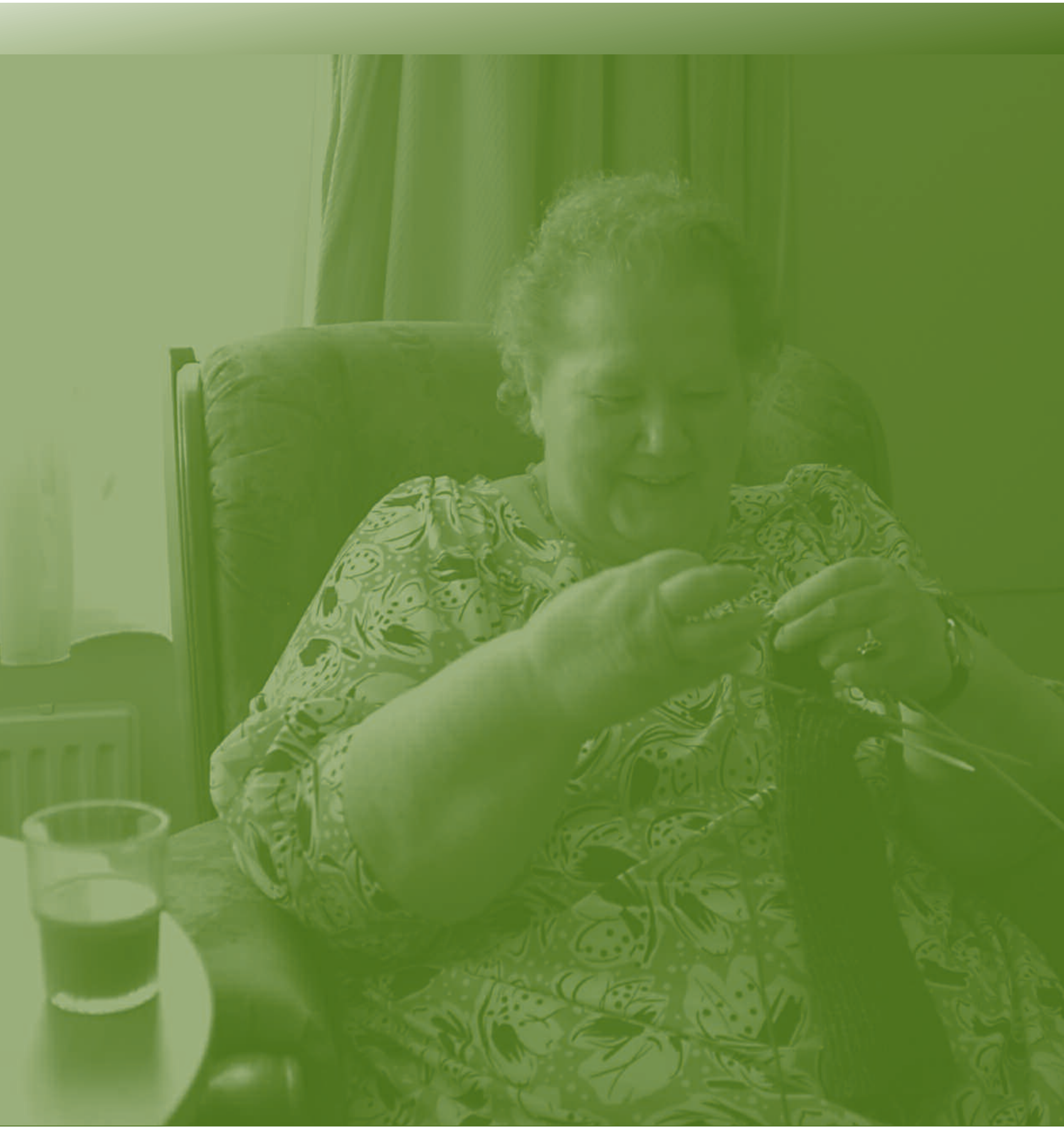

actiz
organisatie van zorgondernemers

V&VN
Beroepsvereniging van zorgprofessionals

LOC

STAATSTOEZICHT OP DE VOLKSGEZONDHEID
INSPECTIE VOOR DE GEZONDHEIDSZORG
JE MAINTIENDRAI

Zorgverzekeraars Nederland

NVVA

Sting
landelijke beroepsvereniging
verzorging

Ministerie van
Volksgezondheid,
Welzijn en Sport
