# Supplementary material for: Measuring client experiences in long-term care in the Netherlands: a pilot study with the Consumer Quality Index Long-term Care
Source: BMC Health Serv Res. 2010 Apr 12;10:95. doi: 10.1186/1472-6963-10-95 (PMC2907762; doi:10.1186/1472-6963-10-95)
Supplement: Additional file 2 — Public version of the report 'The tone is set'. Results of the assessments in 2007 and 2008 are shown in this brochure on the report 'The tone is set: a common language for quality.' [file 1472-6963-10-95-S2.PDF]

ZICHTBARE ZORG VERPLEGING VERZORGING THUISZORG

The tone is set: a common language for quality

Public version of the report published by the Quality Framework for Responsible Residential and Domiciliary Care (VV&T) Steering Committee.

• 'Responsible' care – what does that entail?

Dutch law requires health care professionals to provide 'responsible' care. For the first time, this concept has now been defined and made measurable by means of a set of performance indicators and standardized assessment methods known as the 'Quality Framework for Responsible Care'. Since September 2008, this system has allowed information about the performance of care providers in the residential and domiciliary (home care) sectors to be published on a special website (www.kiesbeter.nl) and in annual social responsibility reports, thus establishing transparency and accountability.

The Quality Framework for Responsible Care has been developed with the assistance of patient organizations, care providers, professionals, the Health Care Inspectorate (the official regulatory body) and health insurers. As the name suggests, it is concerned with the *quality* of care, this being the most important aspect to all concerned. The key focus is therefore on patient interests in terms of choice and safety. The Quality Framework is not concerned with general or procedural matters such as whether protocols or handbooks are in use, or how many times a week a patient is able to take a shower.

Milestone
The creation of the Quality Framework represents a milestone in the achievement of a common aim: to improve the quality of health care services in the Netherlands yet further. It enables providers to demonstrate their abilities and accomplishments, with full transparency as an important condition.

The patient decides
Every patient is entitled to good and responsible care. Patients' own opinions therefore play a significant part within the Quality Framework. Knowing that their services will be subject to open review encourages care providers to discuss patient requirements more thoroughly and to do even more to ensure satisfaction.

Professional care
The standards of 'responsible' care on which the Quality Framework is based have been developed further to patients' own indications of how their needs can best be met. Patients were also asked about their expectations in terms of the professionalism of the staff they encounter, and the way in which care services are organized. This information enables all concerned to offer *effective* care.

Well organized
A 'market-led' system which focuses patient requirements is extremely relevant in the context of new funding structures such as the 'personal care budget', and will help to enhance the economic efficiency of care services. If the care provider knows patients' wishes, the available resources can be deployed accordingly.

• A win-win situation

The Quality Framework for Responsible Care reveals the current status of quality in residential and domiciliary care (i.e. care at home), both for the sectors as a whole and for the individual organizations working within them.

The Quality Framework looks at ten specific aspects of 'responsible' care. It uses a set of indicators to assess quality within each aspect. A full description of the indicators and the assessment methods used can be found in the full version of the report, *De toon gezet: één taal voor kwaliteit* (in Dutch).

Patient indicators and performance indicators

There are two types of indicator: 'patient indicators' and 'performance indicators'.

Patient indicators represent the views and opinions of patients themselves. How do staff interact with them? Are they consulted enough? Do they enjoy adequate privacy? These indicators are measured twice a year by means of extensive surveys conducted by accredited market research organizations.

Performance indicators are benchmarks which are measured and reported annually by the care providers themselves, according to strict guidelines. For example, nursing homes may be asked to report the number of falls suffered by their patients in the previous year, the incidence of decubitus (bedsores) among their patients, or the number of occasions on which mistakes have been made in administering medication.

One instrument serving several purposes

- The website www.kiesbeter.nl enables (prospective) patients to compare several aspects of care providers' performance by means of a star-based ranking system.

- Care providers and professionals can use the assessment results to monitor the success of their improvement programmes and to establish priorities for further action. They will, of course, do so in close consultation with patient organizations.

- The Quality Framework offers regulatory bodies such as the Health Care Inspectorate (IGZ) and the Dutch Healthcare Authority (NZa) sound information on which to fulfil their statutory responsibilities. All health care providers are now required to include the results of their Quality Framework assessments in their annual reports.

- For health insurers and patient organizations, the information provided by the Quality Framework assessment provides a good starting point for negotiations with care providers with regard to contracting and pricing.

• Sector evaluation: an initial 'snapshot'

The first assessments further to the Quality Framework for Responsible Care were conducted between April 2007 and April 2008. Performance indicators were measured within 1,797 organizations (or departments), while patient indicators were measured for 855 organizations.

Although caution should be exercised in interpreting the results presented in the resulting 'sector evaluation', they do provide a good indication of performance and the quality of the care provided.

Results
The national results of the initial 'reference point' evaluation are given on the following pages. They relate to the care provided to a total of 192,000 patients. The patient indicators were produced with the help of some 35,000 individual patients (or their representatives).

The results presented here are the original 'raw' data. The focus is on improvement. The comments accompanying the indicators therefore list points for attention at the national level.

The results achieved by individual organizations (or departments) are taken from the relevant organizations' annual reports and have been adjusted (standardized) to facilitate comparison. A ranking based on a star system (similar to that used to rate hotels) can be found at www.kiesbeter.nl.

Table 1: General impression based on patient indicators

Indicators	Residential care for patients with physical incapacity	Residential care for psycho-geriatric patients	Domiciliary care
Seen as positive	• Professional and safe practice<br>• Privacy (and living conditions)<br>• Safety of physical environment	• Interaction<br>• Respect for rights; restriction of freedom<br>• Privacy (and living conditions)	• Care plan and evaluation<br>• Physical care and hygiene<br>• Reliability of care staff
Points for attention and/or improvement	• Consultation<br>• Information<br>• Mealtimes and menus	• Consultation<br>• Information<br>• Safety of physical environment	• Consultation<br>• Activities and participation<br>• Coordination and continuity of care

Table 2: General impression based on performance indicators

Indicators	Residential care	Domiciliary care
Positive impression	• Decubitus (bedsores)<br>• Unexpected rapid weight loss (nutrition)<br>• Number of patients receiving influenza vaccination	• Decubitus (bedsores)<br>• Unexpected rapid weight loss (nutrition)<br>• Catheters<br>• Depression
Points for attention and/or improvement	• Number of falls<br>• Number of staff receiving influenza vaccination<br>• Prevalence of urinary incontinence<br>• Diagnosis of cause(s) of urinary incontinence<br>• Depression	• Number of falls<br>• Diagnosis of cause(s) of urinary incontinence

- **Patient opinions**

The results shown in Table 3 represent the scores awarded by patients themselves on a scale of 1 (consistently poor) to 4 (consistently good). Over 80% of the scores average between 3 and 4, which is clearly a positive outcome.

The results are described in greater detail in the 'Sector Evaluation' included in the Steering Committee's full report. A comparison does serve to identify the positive aspects and those in which improvement is required, but as yet the results are not sufficient to establish a national ranking as such. In general, patients receiving care at home report the most positive experiences, followed by (the representatives of) patients in psychogeriatric care.

Notably, 'consultation' and 'information' achieve relatively low scores. It would seem that not enough is being done at the national level to meet patient requirements. However, patients and their representatives are generally satisfied with the level of privacy accorded to them in residential care facilities.

Table 3: Personal experience of patients (or their representatives) expressed on a scale of 1 to 4: average scores (2007)

	Sector		
	**Residential care for patients with physical incapacity**	**Residential care for psycho-geriatric patients**	**Domiciliary care**
Care plan	3.20	3.41	3.64
Consultation	2.65	2.82	2.95
Interaction	3.39	3.47	3.59
Information	2.79	3.29	3.17
Telephone access	-	3.38	3.25
Physical care	3.39	3.18	3.48
Meals and menus	3.00	3.46	-
Professionalism and safety of care	3.44	3.31	3.52
Respect for rights/ restriction of freedom	-	3.51	-
Comfort	3.33	3.26	-
Atmosphere	3.40	3.10	-
Privacy	3.72	3.44	3.46
Activities and participation	3.43	2.97	2.86
Independence/autonomy	3.41	3.09	3.43
Mental well-being	3.20	3.24	3.38
Safety of physical environment	3.72	2.81	3.45
Reliability of staff	-	3.17	3.69
Availability of staff	2.94	2.99	3.31
Coordination and continuity of care	-	-	3.05

Facts and figures

The performance indicators are provided by the care providers themselves, measured using a standardized method. Because the results shown here relate to an initial assessment, there are no norms against which the national percentages can be compared. Nevertheless, certain indicators do lend themselves to comparison with data derived from other sources.

The 'sector evaluation' included in the full report provides more comprehensive information and indicates that a ranking based on the results would be inappropriate at this time. The incidence of decubitus (bedsores) and unexpected weight loss due to malnutrition is, for example, far lower than had previously been thought. However, one rather disappointing finding is that incontinence is not always subject to further investigation by a doctor or specialist nursing practitioner in order to establish cause(s).

Table 4: Availability of standard operational procedures and quality systems

Process indicators	Residential care	Domiciliary care
Have written operational procedures or quality systems been made available? (Number of organizations reporting that they have.) NB These results say nothing about the implementation of the procedures or systems, which may still be in an early stage.		
Demonstrable policy with regard to the prevention of unwarranted restriction of freedom	-	78%
Instruction in use of patient lifts<br>*Can it be shown that staff have indeed been given proper instruction?*	81%	84%
Availability of qualified nurse<br>*Can it be shown that a qualified nurse can always attend a patient within ten minutes?*	81%	-
Availability of doctor<br>*Can it be shown that a doctor can always attend a patient within thirty minutes?*	90%	-
Competence in reserved interventions<br>*Can it be shown that 'reserved' and high-risk interventions are only ever conducted by qualified and competent staff?*	83%	82%

Table 5: Performance indicators: national averages (2007)

Performance indicators	Residential care for psychogeriatric patients	Domiciliary care
Decubitus (bedsores)<br>(% of patients)	3%	1%
Unexpected weight loss<br>(% of patients)	3%	5%
Falls<br>(% of patients per month)	11%	12%
Incidents involving medication<br>(% of patients per month)	8%	-
Antipsychotics, anxiolytics and hypnotics<br>(used at least once per week, % of clients)	42%	-
Antidepressants<br>(used at least once per week, % of patients)	21%	-
Influenza vaccination take-up: patients<br>(% vaccinated patients)	95%	-
Influenza vaccination take-up: staff<br>(% vaccinated patients)	16%	-
Urinary incontinence<br>(several incidents per week; % of patients)	61%	35%
Diagnosis of cause(s) of incontinence (% of affected patients for whom a diagnosis has been confirmed	56%	57%
Catheter<br>(in place for longer than 14 days; % of patients)	4%	4%
Problem behaviour<br>(on one or more occasions in any one week; % of patients)	30%	-
Physical restraint<br>(use required on one or more occasions in any one week, % of patients)	7%*	-
Depression<br>(symptoms of depression lasting three days or longer; % of patients)	24%	16%
Demonstrable policy with regard to the prevention of unwarranted restriction of freedom<br>(demonstrable; % of organizations)	-	78%

** No conclusion can be drawn from this result because the set procedure for measurement/calculation was not followed.*

• A sound basis, but not yet perfect

The initial assessment carried out further to the Quality Framework for Responsible Care provides a wealth of useful information about the quality of complex, long-term care in the residential and home settings. However, it is precisely because this is the initial 'reference' measurement that it would be inappropriate to base any ranking of care organizations on these results at this time.

Further improvement of the system is both possible and desirable. The initial findings may have been influenced by incidental factors, particularly given the relatively small sample size. Moreover, the instructions for measuring performance indicators were not always followed as closely as might be hoped. It is therefore both inappropriate and irresponsible to rank care providers as ‘good’ or ‘bad' based on these results alone. An honest and objective comparison will only be possible when the methodology, both for the measurement itself and the subsequent statistical correction, has been further refined.

Interpretation
A set of 'interpretation rules' has been developed to facilitate the process of establishing and comparing the quality information relating to individual care providers, and is described in detail in the full report. These rules establish the basis for an effective ranking of care organizations. However, it should be remembered that because the Quality Framework remains a 'work in progress', the interpretation rules and ranking model are intended for future rather than immediate use.

Getting better all the time
The announcement of the Quality Framework was preceded by three years of careful preparation. Much was achieved during that time. The Quality Framework is an instrument by which information can be gathered, processed and presented in a manner which is truly unique, not only in the Netherlands but throughout the world.
Although the Quality Framework is not yet a perfect measuring instrument, the possibilities it presents have already been made apparent. It will now be further refined and will eventually develop to become a fully mature, 'grown-up' system which allows the figures at both national level and that of individual care organizations to be monitored over the course of several years. The Steering Committee has listed a number of action points in its report. "Getting better all the time" is a motto which applies equality to the Quality Framework and, more importantly, health care provision itself.

• **Further information**

The report which accompanies this brochure can be downloaded (in Dutch) from:
www.zorgvoorbeter.nl
www.zichtbarezorg.nl

Printed copies can be ordered online at www.zichtbarezorg.nl

Further information about the Quality Framework for Responsible Care can be found at:
www.zorgvoorbeter.nl
www.zichtbarezorg.nl

The results relating to individual health care organizations can be found at:
www.kiesbeter.nl
www.jaarverslagenzorg.nl

This brochure is published by the Quality Framework for Responsible Residential and Domiciliary Care Steering Committee and accompanies the report *De toon gezet: één taal voor kwaliteit* [The tone is set: a common language for quality].

September 2008

actiz organisatie van zorgondernemers	LOC LPR belangenorganisatie cliënten ggz LOC-LPR Zeggenschap in zorg	v&vn Beroepsvereniging van zorgprofessionals
Staatstoezicht op de Volksgezondheid Inspectie voor de Gezondheidszorg Je maintiendrai	Zorgverzekeraars Nederland	Sting landelijke beroepsvereniging verzorging
NVVA	bTn	Ministerie van Volksgezondheid, Welzijn en Sport
